# Fc Effector Function Contributes to the Activity of Human Anti-CTLA-4 Antibodies

**DOI:** 10.1016/j.ccell.2018.02.010

**Published:** 2018-04-09

**Authors:** Frederick Arce Vargas, Andrew J.S. Furness, Kevin Litchfield, Kroopa Joshi, Rachel Rosenthal, Ehsan Ghorani, Isabelle Solomon, Marta H. Lesko, Nora Ruef, Claire Roddie, Jake Y. Henry, Lavinia Spain, Assma Ben Aissa, Andrew Georgiou, Yien Ning Sophia Wong, Myles Smith, Dirk Strauss, Andrew Hayes, David Nicol, Tim O'Brien, Linda Mårtensson, Anne Ljungars, Ingrid Teige, Björn Frendéus, TRACERx Melanoma, Kevin Harrington, Alan Melcher, Andrew Wotherspoon, Nicholas Francis, TRACERx Renal, Ben Challacombe, Archana Fernando, Steve Hazell, Ashish Chandra, Lisa Pickering, Joanna Lynch, Sarah Rudman, Simon Chowdhury, Karen Harrison-Phipps, Mary Varia, Catherine Horsfield, Alexander Polson, Gordon Stamp, Marie O'Donnell, William Drake, Peter Hill, David Hrouda, Eric Mayer, Jonathan Olsburgh, Gordon Kooiman, Kevin O'Connor, Grant Stewart, Michael Aitchison, Maxine Tran, Nicos Fotiadis, Hema Verma, Jose Lopez, TRACERx Lung consortia, Jason Lester, Fiona Morgan, Malgorzata Kornaszewska, Richard Attanoos, Haydn Adams, Helen Davies, Dean Fennell, Jacqui Shaw, John Le Quesne, Apostolos Nakas, Sridhar Rathinam, William Monteiro, Hilary Marshall, Louise Nelson, Jonathan Bennett, Joan Riley, Lindsay Primrose, Luke Martinson, Girija Anand, Sajid Khan, Marianne Nicolson, Keith Kerr, Shirley Palmer, Hardy Remmen, Joy Miller, Keith Buchan, Mahendran Chetty, Lesley Gomersall, Sara Lock, Babu Naidu, Gerald Langman, Simon Trotter, Mary Bellamy, Hollie Bancroft, Amy Kerr, Salma Kadiri, Joanne Webb, Gary Middleton, Madava Djearaman, Yvonne Summers, Raffaele Califano, Paul Taylor, Rajesh Shah, Piotr Krysiak, Kendadai Rammohan, Eustace Fontaine, Richard Booton, Matthew Evison, Phil Crosbie, Stuart Moss, Faiza Idries, Juliette Novasio, Leena Joseph, Paul Bishop, Anshuman Chaturvedi, Anne Marie Quinn, Helen Doran, Angela leek, Phil Harrison, Katrina Moore, Rachael Waddington, Fiona Blackhall, Jane Rogan, Elaine Smith, Caroline Dive, Ged Brady, Dominic Rothwell, Sakshi Gulati, Francesca Chemie, Jonathan Tugwood, Jackie Pierce, David Lawrence, Martin Hayward, Nikolaos Panagiotopoulos, Robert George, Davide Patrini, Mary Falzon, Elaine Borg, Reena Khiroya, Mariam Jamal-Hanjani, Gareth Wilson, Nicolai Juul Birkbak, Thomas Watkins, Nicholas McGranahan, Christopher Abbosh, Stuart Horswell, Richard Mitter, Mickael Escudero, Aengus Stewart, Andrew Rowan, Crispin Hiley, Jacki Goldman, Asia Ahmed, Magali Taylor, Junaid Choudhary, Penny Shaw, Raju Veeriah, Justyna Czyzewska-Khan, Diana Johnson, Joanne Laycock, Robert Hynds, Mariana Werner Sunderland, James Reading, Marco Novelli, Dahmane Oukrif, Sam Janes, Martin Forster, Tanya Ahmad, Siow Ming Lee, Peter van Loo, Javier Herrero, John Hartley, Richard Kevin Stone, Tamara Denner, Marta Costa, Sharmin Begum, Ben Phillimore, Tim Chambers, Emma Nye, Sophie Ward, Greg Elgar, Maise Al-Bakir, Dawn Carnell, Ruheena Mendes, Jeremy George, Neal Navani, Dionysis Papadatos-Pastos, Marco Scarci, Pat Gorman, Helen Lowe, Leah Ensell, David Moore, Mairead MacKenzie, Maggie Wilcox, Harriet Bell, Allan Hackshaw, Yenting Ngai, Sean Smith, Nicole Gower, Christian Ottensmeier, Serena Chee, Benjamin Johnson, Aiman Alzetani, Emily Shaw, Eric Lim, Paulo De Sousa, Monica Tavares Barbosa, Andrew Nicholson, Alex Bowman, Simon Jordan, Alexandra Rice, Hilgardt Raubenheimer, Chiara Proli, Maria Elena Cufari, John Carlo Ronquillo, Angela Kwayie, Harshil Bhayani, Morag Hamilton, Yusura Bakar, Natalie Mensah, Lyn Ambrose, Anand Devaraj, Silviu Buderi, Jonathan Finch, Leire Azcarate, Hema Chavan, Sophie Green, Hillaria Mashinga, Kelvin Lau, Michael Sheaff, Peter Schmid, John Conibear, Veni Ezhil, Vineet Prakash, Sarah Danson, Jonathan Bury, John Edwards, Jennifer Hill, Sue Matthews, Yota Kitsanta, Kim Suvarna, Michael Shackcloth, John Gosney, Pieter Postmus, Sarah Feeney, Julius Asante-Siaw, Peter Russell, Teresa Light, Tracey Horey, Kevin Blyth, Craig Dick, Alan Kirk, Martin Pule, Teresa Marafioti, Martin Gore, James Larkin, Samra Turajlic, Charles Swanton, Karl S. Peggs, Sergio A. Quezada

**Affiliations:** 1 Cancer Immunology Unit, University College London (UCL) Cancer Institute, London WC1E 6DD, UK; 2 Research Department of Haematology, UCL Cancer Institute, London WC1E 6DD, UK; 3 The Royal Marsden NHS Foundation Trust, London SW3 6JJ, UK; 4 Translational Cancer Therapeutics Laboratory, The Francis Crick Institute, London NW1 1AT, UK; 5 Bill Lyons Informatics Centre, UCL Cancer Institute, London WC1E 6DD, UK; 6 Cancer Research UK Lung Cancer Centre of Excellence, UCL Cancer Institute, London WC1E 6DD, UK; 7 Guy's and St Thomas' NHS Foundation Trust, London SE1 9RT, UK; 8 BioInvent International AB, 223 70 Lund, Sweden; 9 Department of Cellular Pathology, University College London Hospital, London NW1 2BU, UK; 10 Translational Cancer Therapeutics Laboratory, UCL Cancer Institute, London WC1E 6DD, UK

**Keywords:** CTLA-4, regulatory T cell depletion, ipilimumab, tremelimumab, antibody-dependent cell-mediated cytotoxicity, Fc-gamma receptors, IgG subclass, immune checkpoints, tumor immunotherapy, immune regulatory antibodies

## Abstract

With the use of a mouse model expressing human Fc-gamma receptors (FcγRs), we demonstrated that antibodies with isotypes equivalent to ipilimumab and tremelimumab mediate intra-tumoral regulatory T (Treg) cell depletion *in vivo*, increasing the CD8^+^ to Treg cell ratio and promoting tumor rejection. Antibodies with improved FcγR binding profiles drove superior anti-tumor responses and survival. In patients with advanced melanoma, response to ipilimumab was associated with the CD16a-V158F high affinity polymorphism. Such activity only appeared relevant in the context of inflamed tumors, explaining the modest response rates observed in the clinical setting. Our data suggest that the activity of anti-CTLA-4 in inflamed tumors may be improved through enhancement of FcγR binding, whereas poorly infiltrated tumors will likely require combination approaches.

## Highlights

• Anti-CTLA-4 of hIgG1 and hIgG2 isotypes promote depletion of intra-tumoral Treg cells
• hIgG2 antibodies mediate *in vivo* depletion of intra-tumoral Treg cells via CD32a
• Anti-CTLA-4 with enhanced Fc effector function improves therapeutic outcomes
• The CD16-V158F SNP is associated with response to ipilimumab in inflamed tumors

## Graphical Abstract

**Figure:**
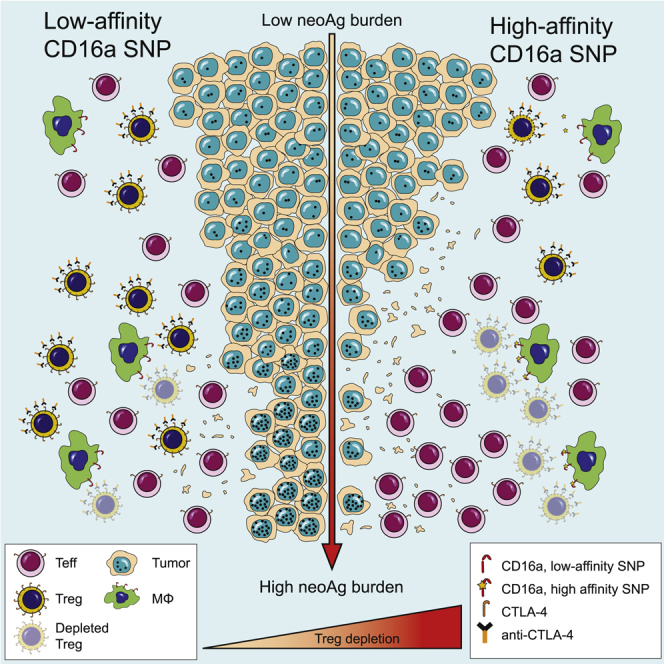

## Teaser

Arce Vargas et al. use a mouse model expressing human FcγRs to show that antibodies with isotypes equivalent to ipilimumab increase the CD8^+^ to Treg ratio by depleting intra-tumoral Tregs to promote tumor rejection. In melanoma patients, response to ipilimumab is associated with a high affinity FcγR polymorphism.

## Significance

**Understanding the mechanisms underlying the activity of antibodies that modulate immune checkpoints is fundamental. This study demonstrates that the activity of anti-CTLA-4 antibodies depends, at least in part, on the depletion of tumor-infiltrating regulatory T (Treg) cells in the context of human FcγRs and human IgGs. Enhanced antibody-dependent cell-mediated cytotoxicity, either by Fc optimization, or the presence of FcγR variants with high binding affinity, improves therapeutic outcomes, but only in highly immunogenic tumors. The combination of mutational burden and FcγR polymorphism status should be considered in the selection of patients likely to respond to anti-CTLA-4. The same rules may apply to the design of immune modulatory antibodies directed against additional targets with high relative expression on Treg cells.**

## Introduction

Modulation of co-inhibitory and co-stimulatory immune checkpoint molecules on tumor-reactive lymphocytes has emerged as a promising therapeutic strategy for a variety of cancers (Hodi et al., 2010, Larkin et al., 2015, Ribas et al., 2015, Robert et al., 2011, Robert et al., 2014, Robert et al., 2015, Weber et al., 2015, Wolchok et al., 2013). Monoclonal antibodies (mAbs) targeting immune checkpoint molecules were initially thought to act solely via regulation of effector T (Teff) cell responses, but recent pre-clinical data in mouse models demonstrates that the activity of certain immune modulatory mAbs (such as anti-CTLA-4, -GITR, and -OX40) may extend beyond simple receptor stimulation or blockade, relying upon an additional capacity to deplete regulatory T (Treg) cells by antibody-dependent cell-mediated cytotoxicity (ADCC) (Bulliard et al., 2013, Bulliard et al., 2014, Selby et al., 2013, Simpson et al., 2013).

Anti-CTLA-4 mAbs have been extensively studied in mouse models of cancer, where rejection of established tumors relies upon the impact of anti-CTLA-4 on CD4^+^ and CD8^+^ Teff and on CD4^+^FoxP3^+^ Treg cells (Peggs et al., 2009). Whilst binding of anti-CTLA-4 to Teff and Treg cells serves to promote expansion of both compartments via its immune modulatory activity, high levels of surface CTLA-4 on tumor-infiltrating Treg cells relative to Teff cells promotes preferential depletion of Treg cells at the tumor site, resulting in an increase in the intra-tumoral Teff/Treg cell ratio and tumor rejection (Bulliard et al., 2013, Selby et al., 2013, Simpson et al., 2013). The observed dual activity of anti-CTLA-4 mAbs relies not only upon higher expression of the target molecule on Treg relative to Teff cells at the tumor site but also upon antibody isotype and enrichment of Fc-gamma receptor (FcγR)-expressing innate effector cell subsets with capacity for ADCC within the tumor microenvironment (Simpson et al., 2013).

Ipilimumab, a human IgG1 mAb directed against CTLA-4, mediates durable remissions in patients with advanced melanoma, although such responses are limited to a small subset (Hodi et al., 2010, Robert et al., 2011, Schadendorf et al., 2015). Despite its potentially depleting isotype, the contribution of ADCC and role of FcγRs in the activity of ipilimumab *in vivo* remains unclear. Two recent clinical studies have identified a reduction in tumor-infiltrating Treg cells after ipilimumab therapy (Romano et al., 2015, Tarhini et al., 2014). Moreover, *in vitro* studies demonstrate that ipilimumab depletes CTLA-4-expressing Treg cells in the presence of FcγR-expressing monocytes and natural killer (NK) cells, consistent with predicted binding affinity for activatory FcγRs (Jie et al., 2015, Romano et al., 2015). A second anti-CTLA-4 mAb, tremelimumab, has also displayed activity in early phase studies (Comin-Anduix et al., 2016). In contrast to ipilimumab, a human IgG2 isotype was selected during the pre-clinical design of tremelimumab to minimize potential ADCC activity (Hanson et al., 2004), thus arguing against a role for Treg cell depletion in the activity of anti-CTLA-4 mAbs in humans.

Perhaps the strongest evidence for a role of FcγR-mediated effector function in antibody-based cancer therapies derives from clinical studies demonstrating an association between clinical responses and specific alloforms of activating hFcγRs. Single-nucleotide polymorphisms (SNPs) in *FCGR2A* (H131R) and *FCGR3A* (V158F) have been associated with improved outcomes owing to a higher binding affinity to IgG1 and IgG2, which increases ADCC (Cartron et al., 2002, Musolino et al., 2008, Weng and Levy, 2003, Zhang et al., 2007). However, there has been no formal assessment of the impact of such polymorphisms on the response to anti-CTLA-4 or other immune modulatory mAbs.

Deciphering the contribution of the antibody fragment crystallizable (Fc)-FcγR interaction to the activity of immune modulatory antibodies has the potential to significantly inform the optimal design of the next generation of therapeutics. Mutagenesis and glycoform engineering of mAbs have been demonstrated to modulate the affinity of Fc-FcγR interaction, with impact upon cytotoxicity in cell-based assays (Duncan et al., 1988, Redpath et al., 1998, Sarmay et al., 1992, Shields et al., 2001, Shields et al., 2002). In this context, efficacy studies in mouse models represent an important step in the pre-clinical development of antibody-based therapies. However, reliable translation of such findings across species is often problematic owing to variation in FcγR subtypes, their distribution, and the affinity of individual IgG subclasses in each species. In addition, polymorphisms in human FcγRs may further influence the binding and biological effects of different IgG subtypes (Koene et al., 1997, Warmerdam et al., 1991, Wu et al., 1997), but their potential contribution to the activity of immune modulatory antibodies has not been explored. Here we sought to determine the contribution of Treg cell depletion to the *in vivo* anti-tumor activity of anti-CTLA-4 antibodies in the context of human FcγRs and human IgG isotypes.

## Results

### CTLA-4, GITR, ICOS, and OX40 Are Expressed at Highest Density on Tumor-Infiltrating Treg Cells in Mouse and Human

**Figure 1:**
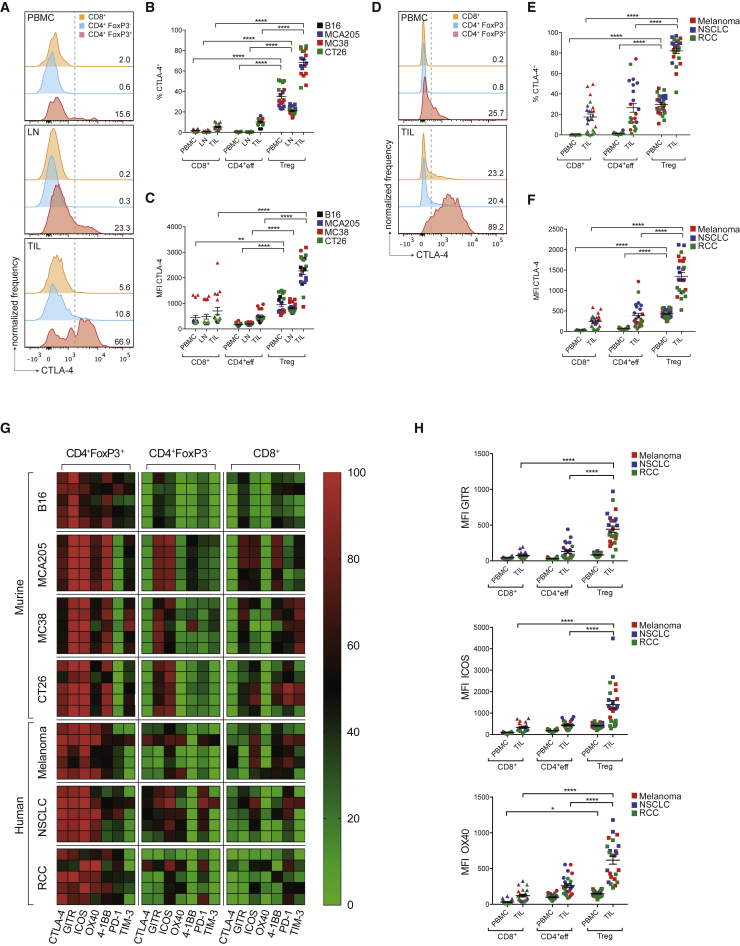
CTLA-4, GITR, ICOS and OX40 Are Highly Expressed by Tumor-Infiltrating Treg Cells (A–C) Mice (n = 5) were injected subcutaneously (s.c.) with B16, MCA205, MC38 (C57BL/6 mice) or CT26 (Balb/c mice) cells. Ten days later, cell suspensions of PBMC, draining LNs and tumor-infiltrating lymphocytes (TILs) were stained and analyzed by flow cytometry. (A) Representative histograms of CTLA-4 expression detected by intracellular staining of individual T cell subsets in mice with MCA205 tumors. Dotted lines represent the gates, numbers indicate the percentage of CTLA-4^+^ cells. (B and C) Percentage (B) and MFI (C) of CTLA-4-expressing cells in murine PBMCs, LNs, and TILs in different tumor models. (D) Representative histograms of CTLA-4 expression detected by intracellular staining of T cell subsets in PBMCs and TILs in a patient with advanced melanoma. (E and F) Percentage (E) and MFI (F) of CTLA-4 expression in T cells in PBMCs and TILs of patients with advanced melanoma (n = 8), early-stage NSCLC (n = 8) and RCC (n = 8). (G) Heatmap demonstrating the percentage of cells expressing co-inhibitory and co-stimulatory molecules within the indicated T cell subsets quantified by flow cytometry. Each row represents an individual murine or human tumor (n = 5). (H) MFI of the indicated co-inhibitory and co-stimulatory molecules in PBMCs and TILs in patients with melanoma. Horizontal bars represent the mean; error bars show ± standard error of the mean (SEM). ^∗^p < 0.05; ^∗∗∗∗^p < 0.0001. See also Table S1 and Figure S1.

CTLA-4 has been described to be constitutively expressed on Treg cells (Read et al., 2000, Read et al., 2006, Wing et al., 2008) and emerging data suggest this may also be relevant to Treg cells infiltrating human tumors (De Simone et al., 2016, Plitas et al., 2016). We sought to comprehensively evaluate the relative expression of CTLA-4 on circulating and tumor-infiltrating CD4^+^FoxP3^+^, CD4^+^FoxP3^−^, and CD8^+^ T lymphocytes across multiple murine models of transplantable syngeneic tumor cell lines of variable immunogenicity, including B16 melanoma, MCA205 sarcoma, MC38 colonic adenocarcinoma, CT26 colorectal carcinoma (Figures 1A–1C), and human solid tumor subtypes including advanced melanoma, early-stage non-small cell lung cancer (NSCLC), and renal cell carcinoma (RCC) (Figures 1D–1F). In mice, CTLA-4 expression was evaluated in peripheral blood mononuclear cells (PBMCs), draining lymph nodes (LNs), and tumor-infiltrating lymphocytes (TILs) by flow cytometry 10 days after tumor challenge. In humans, PBMCs and tumor digests were isolated from blood and resection specimens at matched time points (Table S1).

Across all studied mouse models, CTLA-4 expression appeared higher in the tumor and was largely restricted to CD4^+^FoxP3^+^ Treg cells (mean expression 68.3%), relative to CD4^+^FoxP3^−^ effector (CD4^+^eff) T cells (10.2%, p < 0.0001) and CD8^+^ T cells (5.4%, p < 0.0001) (Figures 1A and 1B). Where CTLA-4 expression was observed on TIL subsets other than Treg cells, this was at significantly lower levels based on mean fluorescent intensity (MFI; mean MFI Treg cells 2,271.8 relative to CD4^+^Teff cells 498.6, p < 0.0001, and CD8^+^ T cells 701.0, p < 0.0001, Figure 1C).

In human tumors, CTLA-4 expression also appeared higher in TILs relative to PBMCs and its expression profile among T cell subsets was similar to mouse models (mean expression in Treg cells 82.1%, relative to CD4^+^eff T cells 26.5%, p < 0.0001 and CD8^+^ T cells 17.4%, p < 0.0001, Figures 1D and 1E). Although CTLA-4 expression was also observed in a proportion of human CD4^+^eff and CD8^+^ TILs, this was again at significantly lower levels based on MFI (mean MFI Treg cells 1,349.6 relative to CD4^+^eff T cells 385.9, p < 0.0001 and CD8^+^ T cells 239.4, p < 0.0001, Figure 1F). CTLA-4 was consistently expressed at low levels on CD8^+^ T cells within tumors, with a mean MFI lower than that observed among tumor-infiltrating and circulating Treg cells in mouse models and human tumors (Figures 1C and 1F).

Based on these data, we sought to determine the expression of an extended panel of immune checkpoint molecules of B7 and tumor necrosis factor receptor superfamilies on TIL subsets (Figures 1G and 1H). Significant heterogeneity in expression profiles was observed between different tumor subtypes, particularly in molecules typically described on Teff cells, including 4-1BB, PD-1, and TIM-3 (Figure 1G). The percentage of cells expressing these molecules appeared higher among CD8^+^ T cells in the more immunogenic MCA205, MC38, and CT26 mouse tumors relative to the poorly immunogenic B16 melanoma and also higher in human melanoma relative to NSCLC and RCC, potentially related to the immunogenic burden of somatic mutations typically associated with these tumor subtypes (Alexandrov et al., 2013).

Despite this, a number of potentially exploitable patterns were observed. Similar to CTLA-4, the co-stimulatory receptors GITR, ICOS, and OX40 were consistently expressed on tumor-infiltrating Treg cells in mouse and human tumors. Although a proportion of CD4^+^FoxP3^−^ and CD8^+^ T cell subsets also expressed these molecules (Figure 1G), the level of expression, based on MFI, was significantly lower than on the Treg cell compartment (Figures 1H and S1A). This is in contrast to the co-inhibitory molecules PD-1 and TIM-3, which were expressed by all studied T cell subsets but at highest levels among CD8^+^ T cells in human cancers (Figure S1B). Based on the differential expression between Treg and Teff cells, CTLA-4, GITR, and OX40 appear to be potential targets in all three human tumor subtypes for dual activity antibodies with capacity for ADCC of intra-tumoral Treg cells. Such findings are consistent with pre-clinical mouse studies, in which depleting isotypes of anti-GITR and anti-OX40 demonstrated maximal anti-tumor activity *in vivo*, associated with their ability to enhance effector function with concomitant depletion of tumor-infiltrating Treg cells (Bulliard et al., 2014, Coe et al., 2010).

### Expression Pattern of FcγRs in Human FcγR Mice and Human Tumors

Beyond distribution and density of target molecule expression, the final outcome of antibody-based therapies also depends upon effector function mediated by Fc-FcγR interaction (Furness et al., 2014). FcγR-dependent mechanisms identified in mouse models are not easily translated to the human setting owing to inter-species variation in FcγR subtypes, expression patterns, and affinity to IgG subclasses. We therefore sought to overcome such challenges with use of a mouse model described to recapitulate human FcγR (hFcγR) structural and functional diversity (Smith et al., 2012), comparing FcγR expression profiles with human melanoma in an attempt to validate its translational value.

**Figure 2:**
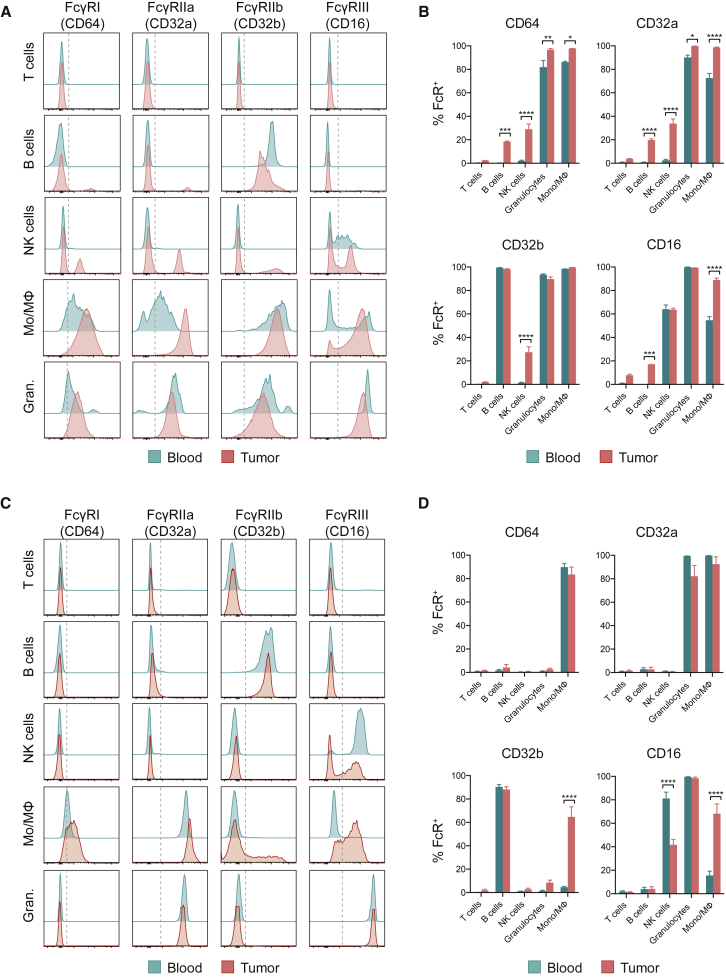
Expression Pattern of FcγRs in hFcγR Mice and Human Tumors The expression of hFcγRs was analyzed by flow cytometry in leukocyte suspensions obtained from blood and MCA205 tumors in hFcγR mice and from metastatic deposits of human melanoma and paired blood samples. (A) Representative histograms demonstrating FcγR expression on CD3^+^ T cells, CD19^+^ B cells, NK1.1^+^ NK cells, CD11b^+^NK1.1^−^Ly6G^−^CD11c^low/−^ monocyte/macrophages (Mo/MΦ) and CD11b^+^Ly6G^+^ granulocytes isolated from hFcγR mice 10 days after s.c. tumor inoculation. (B) Percentage of expression of FcγRs in hFcγR mice from (A) (n = 3). Results are representative of three independent experiments. (C) Representative histograms demonstrating FcγR expression on CD3^+^CD56^−^ T cells, CD19^+^CD3^−^ B cells, CD56^+^CD3^−^ NK cells, CD11b^+^CD14^+^HLA-DR^+^ Mo/Mϕ and CD11b^+^CD15^+^CD14^−^ granulocytes isolated from melanoma patient samples. (D) Percentage expression of FcγRs in metastatic deposits of human melanoma from (B) (n = 10). Error bars show ±SEM. ^∗^p < 0.05; ^∗∗^p < 0.01; ^∗∗∗^p < 0.001; ^∗∗∗∗^p < 0.0001. See also Figure S2 and Table S2.

Analysis of cell subsets in draining LNs, spleens, and blood 10 days after subcutaneous inoculation of MCA205, MC38, or B16 tumors in hFcγR mice demonstrated an expression pattern comparable with previous descriptions (Smith et al., 2012), with activatory FcγRI (CD64), IIa (CD32a), and IIIa/b (CD16a/b) expressed on monocytic and granulocytic myeloid cells, CD16a additionally detected in a fraction of NK cells, and the inhibitory CD32b present on B cells and myeloid cell subpopulations (Figures 2A and 2B and data not shown). Although this expression pattern was maintained on tumor-infiltrating leukocytes, the expression levels of all activatory FcγRs appeared higher in the tumor relative to secondary lymphoid organs, particularly on myeloid cells, which were the most abundant leukocyte subpopulation present in murine tumors (Figure S2A). This pattern was consistent across all three studied tumor models, although the percentage of expression of CD32a and CD16 appeared lower on innate effector cells in B16 tumors relative to the more immunogenic MC38 and MCA205 models (Figure S2B). Of relevance, the absolute number of tumor-infiltrating leukocytes varied between models, with B16 tumors harboring the lowest levels of T cells and innate effector cells relative to MCA205 and MC38 (Figure S2A).

Analysis of human melanoma metastases derived from varied anatomical sites, including subcutaneous, LN, and colonic lesions (Table S2), demonstrated consistent FcγR expression profiles on individual cell subsets, but important differences between tumor and blood (Figures 2C and 2D). FcγR expression on lymphocytes in blood and tumor was confined to CD19^+^CD3^−^ B cells, which expressed the inhibitory receptor CD32b. Activatory FcγR expression was observed on tumor-infiltrating CD56^+^CD3^−^ NK cells, CD11b^+^CD14^+^HLA^−^DR^+^ monocyte/macrophages (Mo/MΦs), and CD11b^+^CD15^+^CD14^−^ granulocytes. In contrast to tumor-infiltrating Mo/MΦs and granulocytes, NK cells accounted for a small fraction of CD45^+^ tumor-infiltrating cell subsets (data not shown). Moreover, where NK cells were identified, expression of CD16a appeared consistently lower on tumor-infiltrating subsets (mean percentage of CD16^+^ in tumor 41.6% versus blood 81.1%, p < 0.05, Figure 2D). Mo/MΦs expressed all three activatory FcγRs (CD64, CD32a, and CD16) as well as the inhibitory receptor CD32b. Although FcγR distribution remained similar between circulating and tumor-infiltrating Mo/MΦs, all FcγRs, particularly CD32b, were consistently expressed at higher levels on tumor-infiltrating Mo/MΦs (Figure 2D). In contrast, FcγR expression by circulating and tumor-infiltrating granulocytes appeared similar, with constitutive expression of the activatory receptors CD32a and CD16b (Figure 2D). Overall, among all tumor-infiltrating leukocyte subsets, CD32a was the most abundantly expressed FcγR in human tumors and highly expressed in mouse tumors (Figure S2C).

FcγR expression in hFcγR mice therefore appeared largely comparable with human melanoma, apart from the inhibitory CD32b. As previously described (Smith et al., 2012), in the mouse model, CD32b was expressed on circulating B cells and on myeloid cells, whereas, in humans, expression in blood was largely confined to B cells. This could result in a less favorable activatory to inhibitory (A:I) FcγR ratio in secondary lymphoid organs in the mouse model relative to human blood and tumors, thus a lack of ADCC activity in these organ sites might not necessarily be reflective of the periphery in humans. However, given the previously demonstrated requirement for activatory rather than inhibitory FcγRs in the activity of anti-CTLA-4 mAbs (Bulliard et al., 2013, Simpson et al., 2013) and the observation that anti-CTLA-4-mediated Treg cell depletion is confined to the tumor site, this was considered less relevant and the model taken forward for *in vivo* studies.

### Human IgG1 and IgG2 Anti-CTLA-4 Antibodies Induce FcγR-Dependent Cytotoxicity *In Vitro*

Based on the comparable expression profile of CTLA-4 on T lymphocytes and FcγRs on tumor-infiltrating innate effector cell subsets in humans and hFcγR mice, we next evaluated whether anti-CTLA-4 mAbs of a human isotype promoted depletion of intra-tumoral Treg cells *in vivo* in a similar manner to that mediated by murine FcγRs (Selby et al., 2013, Simpson et al., 2013).

**Figure 3:**
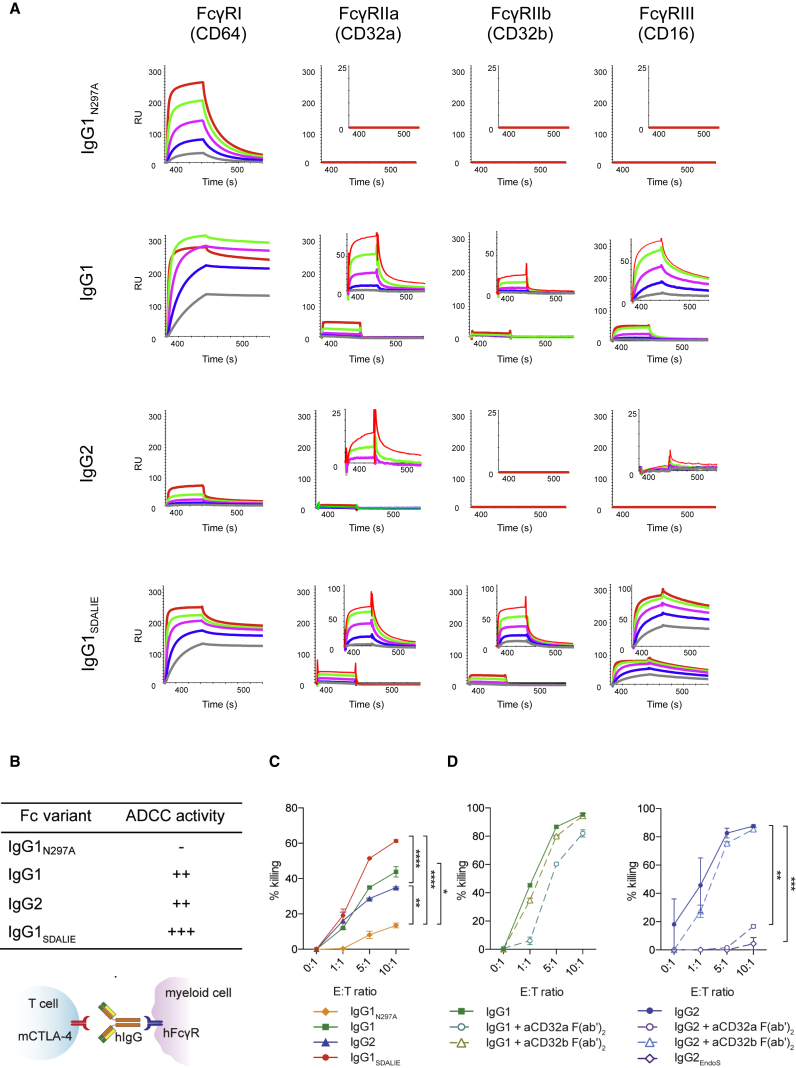
Anti-CTLA-4 Antibodies of IgG1 and IgG2 Isotype Mediate Depletion of CTLA-4-Expressing Target Cells *In Vitro* (A) SPR analysis of anti-murine CTLA-4 with human IgG variants. Large graphs demonstrate interaction of free monomeric FcγRs at increasing FcγR concentrations with immobilized IgG variants; inset graphs show interaction of immobilized IgG variants with aggregated low-affinity FcγRs at increasing concentrations. RU, response units. (B) Schematic representation of the mechanism of action of chimeric anti-mCTLA-4 antibodies and predicted ADCC activity for each human IgG variant. (C) *In vitro* ADCC assay with human monocyte-derived macrophages and mCTLA-4^+^ target cells in the presence of anti-mCTLA-4 mAbs with different human IgG variants. (D) ADCC assay in the presence of CD32a or CD32b-blocking F(ab’)_2_ antibody fragments and with a deglycosylated IgG2 mAb (IgG2_EndoS_). Results are representative of three independent experiments. Error bars show ±SEM of experimental triplicates. ^∗^p < 0.05; ^∗∗^p < 0.01; ^∗∗∗^p < 0.001; ^∗∗∗∗^p < 0.0001. See also Figure S3.

We therefore constructed chimeric anti-murine CTLA-4 (mCTLA-4) antibodies (based on clone 4F10) with human IgG1, modeling ipilimumab, which has been shown to mediate ADCC *in vitro* (Romano et al., 2015). Owing to the abundance of CD32a, the main receptor to which human IgG2 binds, in mouse and human tumors, we also generated a chimeric anti-mCTLA mAb with IgG2, the isotype employed in tremelimumab. These mAbs were compared with mutated IgG1 isotypes with either enhanced binding affinity to activatory CD16a (IgG1_SDALIE_) (Lazar et al., 2006) or no binding to hFcγRs (IgG1_N297A_). Consistent with existing data (Bruhns et al., 2009), surface plasmon resonance (SPR) analysis of generated antibodies demonstrated binding of IgG1 and IgG1_SDALIE_ to all four subtypes of hFcγRs, with a modest increase in the binding affinity of cross-linked IgG1_SDALIE_ relative to wild-type IgG1. IgG2 displayed low binding affinity to activatory CD32a alone, but importantly there was no binding to the inhibitory CD32b, whereas the mutant IgG1_N297A_ demonstrated no binding to any low-affinity hFcγRs (Figures 3A and 3B).

We first assessed their capacity to deplete CTLA-4-expressing target cells *in vitro* in the presence of monocyte-derived human macrophages at varying effector to target (E:T) cell ratios (Figure 3C). As predicted, based on affinity for FcγRs expressed on monocyte-derived human macrophages (Figure S3), which mirrored human melanoma, the IgG1 and IgG2 mAbs demonstrated superior ADCC activity relative to IgG1_N297A_. Furthermore, the IgG1_SDALIE_ mAb, which has an optimized A:I FcγR-binding ratio, promoted enhanced ADCC activity relative to all evaluated isoforms at E:T ratios of 5:1 and above. IgG2-mediated depletion appeared CD32a dependent, as previously described (Schneider-Merck et al., 2010), with loss of activity upon CD32a blockade or use of an Fc-silent deglycosylated form of IgG2 (IgG2_EndoS_, Figure 3D).

### Intra-tumoral Treg Cell Depletion Underlies the Activity of Human IgG1 and IgG2 Anti-CTLA-4 Antibodies

**Figure 4:**
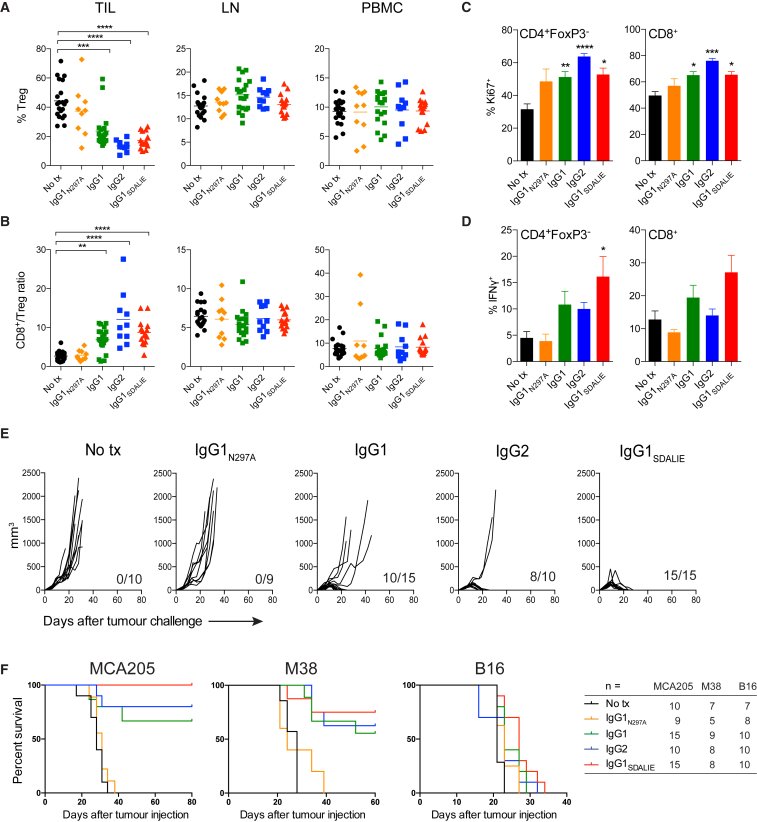
Intra-tumoral Treg Cell Depletion Is Required for the Anti-tumor Activity of Anti-CTLA-4 Mice were treated with 200 μg of anti-CTLA-4 on days 6 and 9 after s.c. inoculation of MCA205 tumor cells (n = 9–21). TILs, LNs, and PBMCs were processed on day 11 and stained for flow cytometry analysis. (A) Percentage of FoxP3^+^CD4^+^ Treg cells from total CD4^+^ T cells. (B) CD8^+^/Treg cell ratio in the indicated sites. Horizontal bars represent the mean. (C) Percentage of Ki67-expressing CD4^+^FoxP3^−^ and CD8^+^ T cells. (D) Percentage of CD4^+^FoxP3^−^ and CD8^+^ T cells expressing IFNγ following re-stimulation with phorbol 12-myristate 13-acetate (PMA) and ionomycin; cumulative data of three separate experiments. Error bars show ±SEM. (E and F) hFcγR mice were treated with anti-CTLA-4 on days 6, 9, and 12 after s.c. inoculation of MCA205 (50 μg/dose), MC38 (100 μg/dose) or B16 (200 μg/dose) tumor cells. (E) MCA205 tumor growth in individual hFcγR mice in each treatment group. Inset numbers show the fraction of mice with complete long-term response. (F) Kaplan-Meier curves demonstrating survival of hFcγR mice for each tumor model. The total number of mice in each treatment group is shown at the right. ^∗^p < 0.05; ^∗∗^p < 0.01; ^∗∗∗^p < 0.001; ^∗∗∗∗^p < 0.0001. See also Figure S4.

We next sought to determine the impact of chimeric anti-mCTLA-4 IgG variants *in vivo* in hFcγR mice. This was purposefully evaluated in the MCA205 model to analyze Treg cell depletion in the context of an inflamed tumor (Figure 4A). Consistent with *in vitro* data, there was a reduction in the proportion of tumor-infiltrating Treg cells in mice treated with the IgG1 mAb (mean percentage of Treg/total CD4^+^ T cells = 24%) compared with those treated with the IgG1_N297A_ variant (Treg/total CD4^+^ T cells = 37%) or with control mice (Treg/total CD4^+^ T cells = 44%, p <0.001). The depleting activity of the IgG1_SDALIE_ isotype appeared superior to the wild-type IgG1 mAb (Treg/total CD4^+^ T cells 17% versus 24%, respectively), but this did not meet statistical significance. The IgG2 isotype, often described as a poor mediator of ADCC since it only binds to activatory CD32a (Schneider-Merck et al., 2010), efficiently depleted tumor-infiltrating Treg cells *in vivo* (Treg/total CD4^+^ T cells = 13%), with comparable activity to that observed in mice treated with the IgG1 and IgG1_SDALIE_ isotype variants. Similar to *in vitro* observations, this effect was CD32a-dependent and no Treg cell depletion was observed in mice treated with Fc-silent IgG2_EndoS_ mAb or in hFcγR mice lacking expression of CD32a (*FCGR2A*^−/−^) (Figure S4).

As previously described in wild-type mice (Simpson et al., 2013), the depleting activity of all human IgG variants in this model was restricted to the tumor microenvironment, with no impact on Treg cells in LNs or blood (Figure 4A). As a result, anti-CTLA-4 mAb of human IgG1, IgG1_SDALIE_, and IgG2 isotypes led to an increase in the intra-tumoral ratio of CD8^+^ to Treg cells (Figure 4B). This was only observed within the tumor microenvironment, demonstrating that, in the context of human FcγR-human IgG interactions *in vivo*, depletion of tumor-infiltrating Treg cells is a major contributor to the shift in this ratio, which has previously been associated with therapeutic responses in mouse and humans (Hodi et al., 2008, Quezada et al., 2006). Treg cell depletion also correlated with a higher proliferation of CD4^+^eff and CD8^+^ T cells independently of the isotype, although only the IgG1_SDALIE_ mAb resulted in a significantly higher production of interferon-γ (IFNγ) by CD4^+^eff T cells (Figures 4C and 4D).

In order to determine the impact of intra-tumoral Treg cell depletion on anti-tumor activity and survival, hFcγR mice were challenged with subcutaneous MCA205, MC38, or B16 tumors on day 0 and subsequently treated with chimeric anti-mCTLA-4 mAb IgG variants on days 6, 9, and 12. Among MCA205 tumors, growth was equivalent in mice left untreated or in those treated with the Fc-silent IgG1_N297A_ anti-mCTLA-4. This finding was of key relevance, demonstrating that CTLA-4 blockade alone is insufficient to promote tumor rejection in the context of human FcγR-IgG interactions. In contrast, the majority of mice treated with either IgG1 or IgG2 anti-CTLA-4 mAbs rejected tumors completely (66.67% and 80%, respectively). Anti-CTLA-4 IgG1_SDALIE_, with enhanced affinity for activating FcγRs, resulted in eradication of established tumors in all treated mice (Figure 4E). Importantly, responses appeared durable, with responding mice from all treatment groups alive for more than 80 days (Figure 4F).

Similar responses were observed among mice bearing MC38 tumors, where the therapeutic effect, although lower than in MCA205 tumors despite higher doses of mAbs, was only observed in the groups treated with depleting isotypes. Although the proportion of complete responses was higher in the IgG1_SDALIE_ group (75.0%) compared with the IgG1 and IgG2 treatments (66.67% and 62.5%, respectively), these differences were not statistically significant. In contrast, correlating with an observed paucity of both T and innate effector cell infiltration (Figure S2A), anti-CTLA-4 mAbs lacked efficacy against B16 tumors despite the use of a higher dose of antibody and regardless of isotype (Figure 4F).

Our pre-clinical data support a unifying hypothesis in which both hIgG1 and hIgG2 anti-CTLA-4 mAbs employed in the clinic act to promote preferential depletion of tumor-infiltrating Treg cells and increase the intra-tumoral Teff/Treg cell ratio associated with tumor rejection. The observed lack of activity against B16 melanoma indicates that such activity is likely only relevant to inflamed tumors with abundant target molecule expression and FcγR-expressing innate effector cell subsets. Further, our data suggest that optimization of A:I FcγR binding through Fc engineering may promote enhanced Treg cell depletion and anti-tumor activity in this context.

### Human FcγR Polymorphisms Impact Response to Ipilimumab in Patients with Advanced Melanoma

In humans, the strongest evidence for a role of FcγR-mediated effector function in tumor-targeting antibody-based cancer therapies (e.g., rituximab) derives from studies demonstrating an association between clinical responses and specific alloforms of activating FcγRs conferring higher binding affinity to IgG1 or IgG2, particularly the CD16a-V158F and CD32a-H131R SNPs, respectively (Cartron et al., 2002, Musolino et al., 2008, Weng and Levy, 2003, Zhang et al., 2007). However, no association between FcγR polymorphisms and clinical outcome has been described in the context of anti-CTLA-4 or other immune checkpoint modulators.

**Figure 5:**
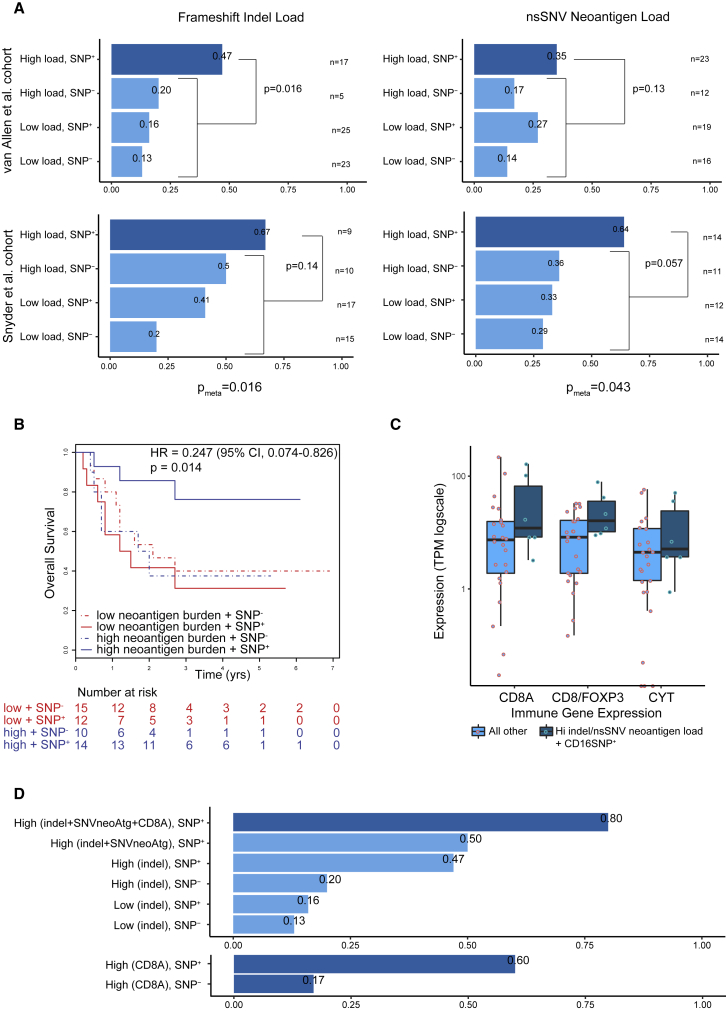
Human FcγR Polymorphisms Impact Response to Ipilimumab in Patients with Advanced Melanoma (A) Anti-CTLA-4 response rate analysis in two separate cohorts of advanced melanoma patients, as published by Van Allen et al. (2015) (top) and Snyder et al. (2014) (bottom). For each analysis patients are split into four groups: (1) high load of somatic mutations and presence of germline high-affinity CD16a-V158F polymorphism (SNP^+^), (2) high load of somatic mutations and absence of germline CD16a-V158F polymorphism (SNP^−^), (3) low load of somatic mutations and SNP^+^, and (4) low load of somatic mutations and SNP^−^. Both homozygous and heterozygous patients were included in the SNP^+^ groups. Two different measures of mutational load were tested (McGranahan et al., 2016, Turajlic et al., 2017): (left) the number of frameshift indel mutations and (right) the number of non-synonymous single-nucleotide variant (nsSNV) neoantigens. In all cases, high and low are defined as above or below the median value, respectively. In each analysis, patient group (1) is tested for a difference in response rate compared with groups 2–4 using Fisher's exact test. Meta-analysis for each measure (p_meta_), across the two patient cohorts was conducted using the Fisher's method of combining p values from independent tests. (B) Survival analysis of patients with advanced melanoma treated with anti-CTLA-4 with low (≤median) or high (>median) predicted neoantigen burden with or without the germline polymorphism CD16a-V158F. Log rank p values are displayed with hazard ratio (HR) and confidence interval (CI). In (A) and (B), patients from the Snyder et al. (2014) cohort treated with tremelimumab (n = 3) were excluded. (C) Boxplot showing the expression level of key immune markers from patients with available RNA-seq data from the Van Allen et al. (2015) cohort (n = 30): *CD8A*, ratio of *CD8A* divided by *FOXP3* and cytolytic activity (defined as the log-average of *GZMA* and *PRF* expression). Patients are grouped into responders with high mutational load (based on either measure) and SNP^+^, compared with all other patients. Boxes show the middle quartile (25%–75%); horizontal bars represent the median; whiskers show either the maximum and minimum values in the dataset or ±1.5 times the interquartile range if the maximum and minimum values exceed these limits. TPM, transcripts per million. (D) Extension of the response rate analysis from (A), top left, with the following additional two groups: high mutational load (for both measures) plus high *CD8A* expression (>median) plus SNP^+^ and high mutational load (for both measures) plus SNP^+^ (top bar graph). In addition, high *CD8A* expression plus SNP^+^ and high *CD8A* expression plus SNP^−^ were compared (bottom bar graph). Due to the small RNA-seq sample size (n = 30), differences were not tested for statistical significance in (C) and (D). See also Figure S5.

Mutational burden and putative neoantigen burden have been identified as predictive markers of response to ipilimumab in patients with advanced melanoma (Van Allen et al., 2015, McGranahan et al., 2016, Nathanson et al., 2016, Snyder et al., 2014), pointing to mutations as a potential substrate for tumor recognition by T cells. More recently, tumor-specific indel mutations (insertion and deletions) have been identified as a highly immunogenic mutational class that can trigger an abundance of neoantigens and greater mutation binding specificity (Turajlic et al., 2017). Since CTLA-4 is thought to be relevant in the context of T cell receptor engagement (Leach et al., 1996), we sought to determine the impact of the CD16a-V158F and CD32a-H131R SNPs, identified through sequencing of germline DNA, on response to ipilimumab in two separate cohorts of patients with advanced melanoma (Van Allen et al., 2015, Snyder et al., 2014) (Figure 5). We hypothesized that response would be associated with higher non-synonymous single-nucleotide neoantigens (nsSNV neoAg) or indel mutational burden (i.e., a substrate T cell response that could be amplified by ipilimumab) and presence of the CD16a-V158F or CD32a-H131R SNP.

Among tumors with low indel burden (≤median), the CD16-V158F polymorphism was not observed to have an impact on response. However, among those with high indel burden (>median), presence of the CD16-V158F SNP was associated with higher rates of response in both Van Allen et al. (2015) and Snyder et al. (2014) datasets (Figure 5A, left graphs). Meta-analysis of both datasets demonstrated significantly higher response rates in those with high indel burden and the CD16-V158F SNP, as compared with all other patients (p = 0.016). Similar findings were observed when considering nsSNV neoantigens and the presence or absence of the CD16-V158F (Figure 5A, right graphs, p = 0.043). Once again, meta-analysis of both datasets demonstrated significantly higher response rates among those with high neoantigen burden (>median) and the CD16-V158F SNP. Further, in the Snyder et al. (2014) dataset, patients with both high neoantigen burden and the CD16-V158F SNP had significantly improved overall survival (p = 0.014, Figure 5B). Although the same trend was observed in the survival analysis of the Van Allen et al. (2015) dataset, the differences were not statistically significant. Such observations were not common to the CD32a-H131R polymorphism, which is associated with greater affinity for IgG2 rather than IgG1 (Figures S5A and S5B) (Parren et al., 1992, Salmon et al., 1992).

Feasibly, improved response rates and survival in patients with the CD16-V158F SNP could also relate to enhancement of other immunological processes mediated by FcγR-expressing cell subsets, including antigen processing and presentation. However, analysis of a cohort of patients with advanced melanoma treated with pembrolizumab or nivolumab (Hugo et al., 2016), both IgG4 mAbs directed against PD-1, with low predicted binding affinity to FcγRs, demonstrated no association between the CD16-V158F SNP and response rates in patients with high indel burden (Figure S5C). Indeed, response rates appeared lower in this setting (although not meeting significance). Intriguingly, the CD16-V158F allele is capable of binding to IgG4, raising the possibility that depletion of PD-1^high^ Teff cells via IgG4-mediated ADCC might underlie inferior response rates in those with high indel burden and CD16-V158F SNP.

Finally, a clinically relevant potential surrogate of mutational burden is the magnitude of the immune infiltrate in the tumor. We therefore interrogated RNA sequencing (RNA-seq) data derived from the Van Allen et al. (2015) cohort and compared the expression of key immune markers in responding patients with high mutational load (based on either indel or putative neoantigen burden) and the CD16-V158F SNP with rest of the cohort. Expression levels of CD8A, cytolytic markers (granzyme A and perforin) as well as the CD8A/FoxP3 ratio (based on gene expression) appeared higher in the group with improved response (Figure 5C). Although the size of the cohort with RNA-seq data available in the Van Allen et al. (2015) dataset (n = 30) was too small to allow adequate statistical analysis, the presence of high indel or putative neoantigen burden, CD8A, and the CD16-V158F SNP was associated with higher response rates than any other combination of metrics (Figure 5D), supporting the hypothesis that, in inflamed or highly infiltrated tumors, anti-CTLA-4 antibodies function, at least in part, via engagement of FcγRs and depletion of Treg cells.

## Discussion

Pre-clinical studies in mouse models of cancer have demonstrated that the activity of certain immune modulatory antibodies may extend beyond receptor stimulation or blockade of Teff cells, relying upon concomitant depletion of Treg cells for maximal anti-tumor activity (Bulliard et al., 2013, Bulliard et al., 2014, Selby et al., 2013, Simpson et al., 2013). Preferential depletion of tumor-infiltrating Treg cells by antibodies targeting CTLA-4, GITR, and OX40 depends upon both a higher density of the target molecule on intra-tumoral Treg cells compared with Teff cells and the presence of an appropriate population of cells to mediate ADCC. Despite the growing body of evidence supporting the premise that immune modulatory antibodies can bear dual (immune modulatory and Treg cell depleting) activity, less evidence exists to support that this mechanism of action is as important in the clinical context.

Here, we extend our previous findings by using hFcγR mice and chimeric anti-mCTLA-4 mAbs with human IgG variants to model the rules of engagement for human FcγRs and human IgGs in the context of immune modulatory mAbs, demonstrating that anti-human CTLA-4 mAbs work, at least in part, through depletion of tumor-infiltrating Treg cells. Anti-CTLA-4 mAbs with the same Fc variants employed in ipilimumab (IgG1) and tremelimumab (IgG2) both induced *in vivo* depletion of tumor-infiltrating Treg cells in the context of human FcγRs. Antibodies engineered to enhance this activity had improved anti-tumor activity, whereas those engineered to lack ADCC capacity demonstrated poor anti-tumor activity. The high expression of CTLA-4 in tumor-infiltrating Treg cells and the presence of innate effector cells expressing high levels of CD16 and CD32a activatory FcγRs both in mouse and humans likely explain the preferential local depletion in the tumor by both antibody isotypes.

A relevant finding was a lack of activity in B16 melanoma. This was associated with a paucity of T cell and innate effector cell infiltration and consequent lack of both target molecule expression and FcγR-expressing cell subsets. This was consistent with the observation that the CD16a-V158F polymorphism was associated with improved response rates in patients with advanced melanoma treated with ipilimumab, but only in the context of high putative neoantigen or indel burden. Taken together, these data provide potential explanation for the modest response rates observed to date with anti-CTLA-4 monotherapy (Schadendorf et al., 2015) and suggest that baseline non-inflamed tumors will require combination approaches which serve to promote immune infiltration. This might also explain the observed synergy with combination anti-CTLA-4 and anti-PD-1 therapy observed in clinical trials (Larkin et al., 2015, Wolchok et al., 2013).

Although the extent of the contribution of ADCC to the activity of ipilimumab and tremelimumab has not been formally tested, our mouse model suggests that it is potentially critical and that further enhancement of ADCC may result in enhanced anti-tumor activity and survival. Where ADCC activity is desirable, the IgG1 isotype is most commonly selected owing to its predicted binding affinity for activating FcγRs. However, the intra-tumoral composition of FcγR-expressing cell subsets is rarely considered, both in terms of the expression of individual FcγRs and their relative abundance. We have demonstrated in murine and human tumors that both the activatory FcγR CD16a and the inhibitory receptor CD32b appeared upregulated on tumor-associated macrophages relative to LN and blood. In keeping with this, IgG1_SDALIE_ mAb, with an optimized A:I (CD16:CD32b) binding profile, demonstrated superior anti-tumor activity relative to all evaluated chimeric anti-CTLA-4 Fc variants. Although not meeting significance, this is likely to be the result of the improved efficacy of intra-tumoral Treg cell depletion observed for IgG1_SDALIE_ relative to wild-type IgG1 and consequent higher production of IFNγ by CD4^+^eff T cells in the tumor.

In contrast to the IgG1 isotype, IgG2 is generally regarded as a poor mediator ADCC owing to a relatively low affinity for activatory FcγRs, particularly CD16 (Bruhns et al., 2009), the principal receptor involved in NK cell-mediated ADCC. However, the Fc effector functions of IgG2 are mediated by CD32a and *in vitro* data demonstrate that IgG2 mAbs mediate effective ADCC via CD32a-expressing myeloid cells (Schneider-Merck et al., 2010). In support of these findings, we demonstrated that chimeric anti-mCTLA-4 IgG2 mAb depletes intra-tumoral Treg cells *in vivo* to a similar extent as the IgG1 mAb. These results may be explained by the relative abundance of CD32a-expressing tumor-infiltrating myeloid cells, which are more abundant than CD16^+^ NK cells both in murine tumors and human melanoma. Furthermore, the binding of IgG2 to inhibitory CD32b is minimal, resulting in a high A:I (CD32a:CD32b) ratio that favors Fc effector function. These results raise the possibility that Treg cell depletion is also relevant to the activity of tremelimumab and that FcγR polymorphisms may contribute to its activity, since the CD32a-H131R polymorphism confers higher relative binding affinity to IgG2 (Bruhns et al., 2009, Sanders et al., 1995, Schneider-Merck et al., 2010).

Further co-stimulatory and co-inhibitory receptors of clinical relevance, specifically GITR, ICOS, and OX40, exhibited similar expression profiles to that of CTLA-4 and may be better targeted with antibodies displaying dual activity. Target molecule density, antibody isotype, and the intra-tumoral composition of FcγR-expressing cell subsets must all be considered in the design of immune modulatory mAbs. Optimal intra-tumoral ADCC activity may depend on CD16a or CD32a binding, depending on which innate effector cells are enriched within the tumor microenvironment. However, ADCC activity only appears relevant in the context of an inflamed tumor microenvironment, and prospective clinical studies should consider exploring the use of polymorphism status and mutational burden to better identify those patients likely to respond to immune modulatory antibodies armed with dual activity, with appropriate stratification to combination regimens that promote tumor infiltration in those with cold tumors at baseline.

## STAR★Methods

### Key Resources Table

**Table:** 

REAGENT or RESOURCE	SOURCE	IDENTIFIER
**Antibodies**		

Anti-histidine Tag	R&D Systems	Cat#MAB050; RRID:AB_357353
Anti-HLA-DR-PE (L243)	eBioscience	Cat#12-9952; RRIS:AB_1272164
Anti-human 4-1BB-PE (4B4-1)	BioLegend	Cat#309804; RRID:AB_314783
Anti-human CD11b-PerCP-Cy5.5 (ICRF44)	BioLegend	Cat#301328; RRID:AB_10933428
Anti-human CD11c-BV (3.9)	BioLegend	Cat#301628; RRID:AB_11203895
Anti-human CD14-PE-Cy7 (M5E2)	BD Biosciences	Cat#561385; RRID:AB_10611732
Anti-human CD15-PE (HI98)	BioLegend	Cat#301906; RRID:AB_314198
Anti-human CD16a/b-V500 (3G8)	BD Biosciences	Cat#561394; RRID:AB_10611857
Anti-human CD19-BV785 (HIB19)	BioLegend	Cat#302240; RRID:AB_2563442
Anti-human CD3-BV785 (OKT3)	BioLegend	Cat#317330; RRID:AB_2563507
Anti-human CD3-eVolve605 (OKT3)	eBioscience	Cat#83-0037; RRID:AB_2574691
Anti-human CD32a-FITC (IV.3)	StemCell	Cat#60012FI; RRID:AB_2722545
Anti-human CD32b-AF647 (6G11)	BioInvent	N/A
Anti-human CD4-AlexaFluor700 (OKT4)	eBioscience	Cat#56-0048; RRID:AB_914326
Anti-human CD45-BV650 (HI30)	BioLegend	Cat#304044; RRID:AB_2563812
Anti-human CD56-BV711 (HCD56)	BioLegend	Cat#318336; RRID:AB_2562417
Anti-human CD64-AF700 (10.1)	BD Biosciences	Cat#561188; RRID:AB_10612007
Anti-human CD8-BV510 (SK1)	BD Biosciences	Cat#563919; RRID:AB_2722546
Anti-human CTLA-4-APC (L3D10)	BioLegend	Cat#349908; RRID:AB_10679122
Anti-human FoxP3-PE (PCH101)	eBioscience	Cat#12-4776; RRID:AB_1518782
Anti-human GITR-biotin (DT5D3)	Miltenyi Biotec	Cat#130-092-886; RRID:AB_871554
Anti-human ICOS-APC (C398.4A)	BioLegend	Cat#313510; RRID:AB_416334
Anti-human OX40-PE.Cy7 (ACT35)	BioLegend	Cat#350012; RRID:AB_10901161
Anti-human PD-1-BV605 (EH12.2H7)	BioLegend	Cat#329924; RRID:AB_2563212
Anti-human TIM-3-BV650 (7D3)	BD Biosciences	Cat#565564; RRID:AB_2722547
Anti-I-Ab-biotin (25-9-7)	BioLegend	Cat#114403; RRID:AB_313578
Anti-moue FoxP3-PE (FJL-16s)	eBioscience	Cat#12-5773; RRID:AB_465936
Anti-mouse 4-1BB-biotin (17B-5)	eBioscience	Cat#13-1371; RRID:AB_466603
Anti-mouse CD11b-BUV661 (M1/70)	BD Biosciences	Cat#565080; RRID:AB_2722548
Anti-mouse CD11c BV785 (N418)	BioLegend	Cat#117335; RRID:AB_11219204
Anti-mouse CD19-BUV727 (1D3)	BD Biosciences	Cat#564296; RRID:AB_2716855
Anti-mouse CD3-PECy.7 (145-2C11)	eBioscience	Cat#25-0031; RRID:AB_469571
Anti-mouse CD4 –v500, (RM4-5)	BD Biosciences	Cat#560782; RRID:AB_1937315
Anti-mouse CD4-BUV496 (GK1.5)	BD Biosciences	Cat#564667; RRID:AB_2722549
Anti-mouse CD45-BUV563 (30-F11)	BD Biosciences	Cat#565710; RRID:AB_2722550
Anti-mouse CD5 (53-7.3)	eBioscience	Cat#45-0051; RRID:AB_914332
Anti-mouse CD8-BUV805 (53-6.7)	BD Biosciences	Cat#564920; RRID:AB_2716856
Anti-mouse CD8-BV650 (53-6.7)	BioLegend	Cat#100742; RRID:AB_2563056
Anti-mouse CTLA-4-BV605 (UC10-4B9)	BioLegend	Cat#106323; RRID:AB_2566467
Anti-mouse FoxP3-FITC (FJK-16S)	eBioscience	Cat#53-5773; RRID:AB_763537
Anti-mouse GITR-eFluor450 (DTA-1)	eBioscience	Cat#48-5874; RRID:AB_1944395
Anti-mouse ICOS-PE.Cy7 (C398.4A)	BioLegend	Cat#313519; RRID:AB_10641839
Anti-mouse IFNγ-AlexaFluor488 (XMG1.2)	BioLegend	Cat#505813; RRID:AB_493312
Anti-mouse Ki67-eFluor450 (SolA15)	eBioscience	Cat#48-5698; RRID:AB_11151155
Anti-mouse Ly6G-BV 650 (1A8)	BioLegend	Cat#127641; RRID:AB_2565881
Anti-mouse NK1.1-eFluor450 (PK136)	eBioscience	Cat#48-5941; RRID:AB_2043877
Anti-mouse OX40-biotin (OX86)	BioLegend	Cat#119403; RRID:AB_345419
Anti-mouse PD-1-eFluor450 (RMP1-30)	eBioscience	Cat#48-9981; RRID:AB_11151705
Anti-mouse TIM-3-PE (8B.2C12)	eBioscience	Cat#12-5871; RRID:AB_465978
Anti-NK1.1-AlexaFluor700 (PK136)	eBioscience	Cat#56-5941; RRID:AB_2574505
Purified anti-human CD32a F(ab)’_2_ (2E08)	Bioinvent	N/A
Purified anti-human CD32b F(ab)’_2_ (6G11)	Bioinvent	N/A
Streptavidin-BV605	BioLegend	Cat#405229
Streptavidin-BV650	BioLegend	Cat#405232
Streptavidin-BV711	BioLegend	Cat#405241
Viability dye eFluor780	eBioscience	Cat#65-0856

**Chemicals, Peptides and Recombinant Proteins**		

Chitin magnetic beads	New England Biolabs	Cat#E8036
EndoS, chitin labeled	New England Biolabs	Cat#P0741
Goat anti-human F(ab)’_2_	Jackson Immunoresearch	Cat#109-005-097; RRID:AB_2337540
Ionomycin	Sigma	Cat#I0634
Phorbol 12-myristate 13-acetate (PMA)	Sigma	Cat#P8139
Recombinant human CD16	R&D Systems	Cat#4325-FC
Recombinant human CD32a	R&D Systems	Cat#1330-CD
Recombinant human CD32b	R&D Systems	Cat#1875-CD
Recombinant human CD64	R&D Systems	Cat#1257-FC
Recombinant human M-CSF	Cell Guidance Systems	Cat#GFM8

**Critical Commercial Assays**		

CD14 MicroBeads, human	Miltenyi Biotec	Cat#130-150-201
CellTrace CFSE cell proliferation kit	Life Technologies	Cat#C34554
Ficoll Paque Plus	GE Healthcare	Cat#GE17-1440
Fixation/Permeabilization solution kit	BD Biosciences	Cat#554714
FoxP3/Transcription Factor Staining Buffer Set	eBioscience	Cat#00-5523
Liberase TL	Roche	Cat#05401020001
Recombinant DNase I recombinant	Roche	Cat#000000004716728001

**Experimental Models: Cell Lines**		

Mouse: B16	ATCC	
Mouse: CT26	Gift from M. Pule	N/A
Mouse: MC38	Gift from B. Becher	N/A
Mouse: MCA205	Gift from L. Galluzzi	N/A
Human: SupT1-mCTLA-4	Gift from M. Pule	N/A

**Experimental Models: Organisms/Strains**		

Mice: C57BL/6	Charles River Laboratories	N/A
Mice: Balb/c	Charles River Laboratories	N/A
Mice: C57BL/6 FcRα^-/-^*Fcgr1*^*-/-*^*FCGR1*^*tg*^*FCGR2A*^*R131tg*^*FCGR2B*^*I232tg*^*FCGR3A*^*F158tg*^*FCGRIIIB*^*tg*^	J. V. Ravetch (Smith et al., 2012)	N/A
Mice: C57BL/6 FcRα^-/-^*Fcgr1*^*-/-*^*FCGR1*^*tg*^*FCGR2A*^*R131-/-*^*FCGR2B*^*I232tg*^*FCGR3A*^*F158tg*^*FCGRIIIB*^*tg*^	J. V. Ravetch (Smith et al., 2012)	N/A

**Software and Algorithms**		

FlowJo 10.0.8	Tree Star Inc.	N/A
Prism 6	GraphPad Software Inc.	N/A

**Other**		

Sequence of heavy and light chains variable regions of anti-mouse CTLA-4 antibody clone 4F10	J. A. Bluestone	GenBank accession numbers Heavy chain: MG916976 Light chain: MG916977

### Contact for Reagent and Resource Sharing

Further information and requests for resources and reagents should be directed to and will be fulfilled by the Lead Contact, Sergio A. Quezada (s.quezada@ucl.ac.uk).

### Experimental Model and Subject Details

#### Mice

C57BL/6 and BALB/c mice were purchased from Charles River Laboratories. FcγRα^-/-^ human FcγR transgenic mice of C57BL/6 background (Smith et al., 2012) were kindly provided by J. V. Ravetch (The Rockefeller University, New York, USA) and housed and bred in Charles River Laboratories, U.K. *FCGR2A*^-/-^ mice were derived from the same colony. Experiments were performed with 6-10 week old females randomly assigned to experimental groups. All animal studies were performed under University College London and U.K. Home Office ethical approval and regulations.

#### Cell Lines and Tissue Culture

MCA205 cells were cultured in Dulbecco’s modified Eagle medium (DMEM, Sigma) supplemented with 10% fetal calf serum (FCS, Sigma), 100 U/mL penicillin, 100 μg/mL streptomycin and 2 mM L-glutamine (all from Gibco). MC38, CT26, B16 and SupT1 cells were cultured in Roswell Park Memorial Institute (RPMI) media supplemented as above. A cell line with stable expression of murine CTLA-4 was generated by retroviral transduction of Sup-T1 cells. For generation of human macrophages, monocytes were isolated from healthy donor PBMCs using anti-CD14 microbeads (Miltenyi Biotec) and cultured for 7 days in supplemented RPMI with recombinant human macrophage colony-stimulating factor (M-CSF, 50 ng/mL, Cell Guidance Systems).

#### Human Study Oversight

Presented human data derives from three translational studies, each approved by local institutional review board and Research Ethics Committee (Melanoma - REC no. 11/LO/0003, The Royal Marsden NHS Foundation Trust; NSCLC – REC no.13/LO/1546, University College London Hospital and RCC – REC no. 11/LO/1996, The Royal Marsden NHS Foundation Trust). All were conducted in accordance with the provisions of the Declaration of Helsinki and with Good Clinical Practice guidelines as defined by the International Conference on Harmonization. All patients (or their legal representatives) provided written informed consent before enrolment. Patient demographics are displayed in Tables S1 and S2.

### Method Details

#### Antibody Production

Antibodies were produced in Evitria AG (Switzerland). The sequences of the variable regions of the heavy and light chains of anti-mouse CTLA-4, clone 4F10, were kindly provided by J. A. Bluestone (University of California, San Francisco, U.S.A.) and used to generate chimeric antibodies with the constant regions of human IgG1 and IgG2 heavy chains and κ light chain, as well as the mutated IgG1 variants N297A and S239D/A330L/I332E (SDALIE) (Lazar et al., 2006). IgG2 antibodies were deglycosylated with Endo S endoglycosydase and re-purified with chitin microbeads following the manufacturer’s protocol (New England Biolabs).

#### Surface Plasmon Resonance (SPR)

The binding of different human antibody isotypes or Fc-engineered variants of chimeric anti-CTLA-4 to human FcγRs was evaluated by surface plasmon resonance (SPR) using a Biacore 3000 instrument (GE Healthcare). Goat Anti-human-F(ab)’_2_ antibody (100 μg/ml, Jackson Immunoresearch) was immobilized to a CM5 chip using standard amine coupling with a flow rate of 10 μl/min for 10 min. Anti-CTLA-4 mAbs were diluted to 50 μg/ml and added to the surface for 3 min at 10 μl/min. His tagged human FcgRI, FcgRIIa, FcgRIIb and FcgRIII (R&D Systems) were added at 1500, 500, 167, 56 and 16nM to the surface for 1 min at 30 μl/min followed by 5 min dissociation. After each cycle the surface was regenerated twice with glycine buffer pH 1.5. For experiments with crosslinked Fc receptor, Fc-receptors were pre incubated with anti-histidine antibody (R&D Systems) at a 2:1 molar ratio before addition to the surface at 1500, 500, 167, 56 and 16nM.

#### *In Vitro* ADCC Assay

SupT1 cells expressing murine CTLA-4 were labelled with 5 μM carboxyfluorescein succinimidyl ester (CellTrace CFSE Cell Proliferation Kit, Life Technologies) and co-cultured overnight with human macrophages at the indicated ratios in the presence of the indicated mAbs (10 μg/mL). For blocking of FcγRs, macrophages were incubated with anti-CD32a (clone 2E08, Bioinvent) or anti-CD32b (clone 6G11, Bioinvent) F(ab’)_2_ fragments at 50 μg/mL for 30 min at 37°C before adding the therapeutic antibodies and target cells. The absolute number of CFSE-labelled cells in each condition was then quantified by flow cytometry using a defined number of reference fluorescent beads (Cell Sorting Set-up Beads for UV Lasers, ThermoFisher). The percentage of killing was determined as: 100-(number CFSE^+^ targets treated/number CFSE^+^ targets untreated).

#### Tumor Experiments

Mice were injected subcutaneously in the flank with 5 x 10^5^ MCA205, MC38 or CT26 cells, or 5 x 10^4^ B16 cells re-suspended in 100 μL of phosphate buffer solution (PBS). Therapeutic antibodies were injected intra-peritoneally at the time points and doses detailed in the figure legends. Tumors were measured twice weekly and volumes calculated as the product of three orthogonal diameters. Mice were euthanized when any diameter reached 150 mm. For functional experiments, tissues were harvested and processed as described previously (Simpson et al., 2013).

#### Flow Cytometry

Acquisition was performed with a BD Fortessa X20 and X30 (BD Biosciences). The antibodies and fluorescent labels used for staining are shown in the Key Resources Table. Surface staining was performed on ice with antibodies re-suspended in PBS with 2% FBS and 2 mM EDTA. Intranuclear staining of FoxP3 and Ki67 was performed using the FoxP3 Transcription Factor Staining Buffer Set (eBioscience). For intracellular staining of cytokines, cells were re-stimulated with phorbol 12-myristate 13-acetate (PMA, 20 ng/mL) and ionomycin (500 ng/mL) (Sigma Aldrich) for 4 hours at 37°C in the presence of GolgiPlug (BD Biosciences) and stained using the Cytofix/Cytoperm buffer set (BD Biosciences). Absolute cell numbers were quantified by flow cytometry using fluorescent reference beads (ThermoFisher).

#### Processing of Human Tissue

Tumor samples were digested with Liberase TL (0.3 mg/mL, Roche) and DNAse I (0.2 mg/mL, Roche) at 37°C for 30 minutes, homogenized using gentleMACS (Miltenyi Biotech) and filtered through a 0.7 μm cell mesh. Leukocytes were enriched by gradient centrifugation with Ficoll-paque (GE Healthcare). Isolated live cells were frozen and stored in liquid nitrogen until analysis.

#### Advanced Melanoma Checkpoint Inhibitor Treated Patient Datasets

Genomic and clinical outcome data were obtained from three previously published patient datasets. The first cohort by Van Allen et al. (2015) comprised patients with melanoma treated with anti-CTLA-4 therapy; 70 patients were retained for analysis after excluding samples with lack of accurate copy number or clonality estimation and low sequencing depth. The second cohort by Snyder et al (Snyder et al., 2014) comprised patients with melanoma treated with anti-CTLA-4 therapy; 51 patients were retained for analysis after excluding samples with lack of accurate copy number or clonality estimation, low sequencing depth and n=3 patients treated with tremelimumab. Patient exclusions from the van Allen et al. and Snyder et al. datasets are consistent with our previously described analysis (McGranahan et al., 2016), with the additional exclusion of tremelimumab-treated cases in this report. The third cohort was by Hugo et al. (Hugo et al., 2016) comprised patients with melanoma treated with anti-PD-1; 34 patients were retained for analysis after exclusion of cases in which DNA had been extracted from patient derived cell lines and patients in whom tissue tumor purity was below 20%. Patient exclusions from the Hugo et al. cohorts are consistent with our previously described analysis (Turajlic et al., 2017).

#### Genomic Analyses

Variant calling from previously published cohorts and identification of putative clonal nsSNVs and frameshift indels was performed as described previously (McGranahan et al., 2016, Turajlic et al., 2017). RNA sequencing data for n=30 cases was available from the previously published Van Allen et al. (2015) cohort), and transcripts per million (TPM) values were computed using RSEM (RNA-Seq by Expectation-Maximization). Cytolytic activity was defined as the log-average (geometric mean) of *GZMA* and *PRF* expression. SAMtools mpileup was used to find non-reference positions in tumor and germline samples. VarScan2 somatic used the output to identify somatic and germline variants. Variants were annotated using Annovar16.

### Quantification and Statistical Analysis

Flow cytometry data analysis was performed with FlowJo 10.0.8 (Tree Star Inc.). Statistical analyses were performed in Prism 6 (GraphPad Software, Inc.) or R (www.R-project.org); p values were calculated using Kruskall-Wallis analysis of variance and Dunn’s post-hoc test, unless otherwise indicated in the figure legends (ns = p > 0.05; ^∗^ = p < 0.05; ^∗∗^ = p < 0.01; ^∗∗∗^ = p < 0.001; ^∗∗∗∗^ = p < 0.0001). Analysis of Kaplan-Meier survival curves was performed with use of the log-rank test.

## Consortia

### The TRACERx Melanoma Consortium (TRAcking Cancer Evolution through Therapy (Rx)), in Addition to Those Named in the Author List

Kevin Harrington, Alan Melcher, Andrew Wotherspoon, and Nicholas Francis.

http://tracerx.co.uk/studies/melanoma/

### The TRACERx Renal Consortium (TRAcking Cancer Evolution through Therapy (Rx)), in Addition to Those Named in the Author List

Ben Challacombe, Archana Fernando, Steve Hazell, Ashish Chandra, Lisa Pickering, Joanna Lynch, Sarah Rudman, Simon Chowdhury, Karen Harrison-Phipps, Mary Varia, Catherine Horsfield, Alexander Polson, Gordon Stamp, Marie O'Donnell, William Drake, Peter Hill, David Hrouda, Eric Mayer, Jonathan Olsburgh, Gordon Kooiman, Kevin O'Connor, Grant Stewart, Michael Aitchison, Maxine Tran, Nicos Fotiadis, Hema Verma, and Jose Lopez. http://tracerx.co.uk/studies/renal/

### The TRACERx Lung Consortium (TRAcking Cancer Evolution through Therapy (Rx)), in Addition to Those Named in the Author List

Jason Lester, Fiona Morgan, Malgorzata Kornaszewska, Richard Attanoos, Haydn Adams, Helen Davies, Dean Fennell, Jacqui Shaw, John Le Quesne, Apostolos Nakas, Sridhar Rathinam, William Monteiro, Hilary Marshall, Louise Nelson, Jonathan Bennett, Joan Riley, Lindsay Primrose, Luke Martinson, Girija Anand, Sajid Khan, Marianne Nicolson, Keith Kerr, Shirley Palmer, Hardy Remmen, Joy Miller, Keith Buchan, Mahendran Chetty, Lesley Gomersall, Sara Lock, Babu Naidu, Gerald Langman, Simon Trotter, Mary Bellamy, Hollie Bancroft, Amy Kerr, Salma Kadiri, Joanne Webb, Gary Middleton, Madava Djearaman, Yvonne Summers, Raffaele Califano, Paul Taylor, Rajesh Shah, Piotr Krysiak, Kendadai Rammohan, Eustace Fontaine, Richard Booton, Matthew Evison, Phil Crosbie, Stuart Moss, Faiza Idries, Juliette Novasio, Leena Joseph, Paul Bishop, Anshuman Chaturvedi, Anne Marie Quinn, Helen Doran, Angela leek, Phil Harrison, Katrina Moore, Rachael Waddington, Fiona Blackhall, Jane Rogan, Elaine Smith, Caroline Dive, Ged Brady, Dominic Rothwell, Sakshi Gulati, Francesca Chemie, Jonathan Tugwood, Jackie Pierce, David Lawrence, Martin Hayward, Nikolaos Panagiotopoulos, Robert George, Davide Patrini, Mary Falzon, Elaine Borg, Reena Khiroya, Mariam Jamal-Hanjani, Gareth Wilson, Nicolai Juul Birkbak, Thomas Watkins, Nicholas McGranahan, Christopher Abbosh, Stuart Horswell, Richard Mitter, Mickael Escudero, Aengus Stewart, Andrew Rowan, Crispin Hiley, Jacki Goldman, Asia Ahmed, Magali Taylor, Junaid Choudhary, Penny Shaw, Raju Veeriah, Justyna Czyzewska-Khan, Diana Johnson, Joanne Laycock, Robert Hynds, Mariana Werner Sunderland, James Reading, Marco Novelli, Dahmane Oukrif, Sam Janes, Martin Forster, Tanya Ahmad, Siow Ming Lee, Peter van Loo, Javier Herrero, John Hartley, Richard Kevin Stone, Tamara Denner, Marta Costa, Sharmin Begum, Ben Phillimore, Tim Chambers, Emma Nye, Sophie Ward, Greg Elgar, Maise Al-Bakir, Dawn Carnell, Ruheena Mendes, Jeremy George, Neal Navani, Dionysis Papadatos-Pastos, Marco Scarci, Pat Gorman, Helen Lowe, Leah Ensell, David Moore, Mairead MacKenzie, Maggie Wilcox, Harriet Bell, Allan Hackshaw, Yenting Ngai, Sean Smith, Nicole Gower, Christian Ottensmeier, Serena Chee, Benjamin Johnson, Aiman Alzetani, Emily Shaw, Eric Lim, Paulo De Sousa, Monica Tavares Barbosa, Andrew Nicholson, Alex Bowman, Simon Jordan, Alexandra Rice, Hilgardt Raubenheimer, Chiara Proli, Maria Elena Cufari, John Carlo Ronquillo, Angela Kwayie, Harshil Bhayani, Morag Hamilton, Yusura Bakar, Natalie Mensah, Lyn Ambrose, Anand Devaraj, Silviu Buderi, Jonathan Finch, Leire Azcarate, Hema Chavan, Sophie Green, Hillaria Mashinga, Kelvin Lau, Michael Sheaff, Peter Schmid, John Conibear, Veni Ezhil, Vineet Prakash, Sarah Danson, Jonathan Bury, John Edwards, Jennifer Hill, Sue Matthews, Yota Kitsanta, Kim Suvarna, Michael Shackcloth, John Gosney, Pieter Postmus, Sarah Feeney, Julius Asante-Siaw, Peter Russell, Teresa Light, Tracey Horey, Kevin Blyth, Craig Dick, and Alan Kirk. http://tracerx.co.uk/studies/lung/

## Supplemental Information

Document S1. Figures S1–S5 and Tables S1 and S2

Document S2. Article plus Supplemental Information
