# Supplementary material for: Fc Effector Function Contributes to the Activity of Human Anti-CTLA-4 Antibodies
Source: Cancer Cell. 2018 Apr 9;33(4):649–663.e4. doi: 10.1016/j.ccell.2018.02.010 (PMC5904288; doi:10.1016/j.ccell.2018.02.010)
Supplement: Document S1. Figures S1–S5 and Tables S1 and S2 [file mmc1.pdf]

Cancer Cell, Volume *33*

Supplemental Information

Fc Effector Function Contributes to the Activity of Human Anti-CTLA-4 Antibodies

Frederick Arce Vargas, Andrew J.S. Furness, Kevin Litchfield, Kroopa Joshi, Rachel Rosenthal, Ehsan Ghorani, Isabelle Solomon, Marta H. Lesko, Nora Ruef, Claire Roddie, Jake Y. Henry, Lavinia Spain, Assma Ben Aissa, Andrew Georgiou, Yien Ning Sophia Wong, Myles Smith, Dirk Strauss, Andrew Hayes, David Nicol, Tim O'Brien, Linda Mårtensson, Anne Ljungars, Ingrid Teige, Björn Frendéus, TRACERx Melanoma, TRACERx Renal, TRACERx Lung consortia, Martin Pule, Teresa Marafioti, Martin Gore, James Larkin, Samra Turajlic, Charles Swanton, Karl S. Peggs, and Sergio A. Quezada

Table S1, related to Figure 1. Demographics and clinical characteristics of patients with advanced malignant melanoma (MM), non-small cell lung cancer (NSCLC) and renal cell carcinoma (RCC).

Identifier	Age	Sex	Subtype	Stage	Biopsy/ resection site	Mutational status	Current therapy	Previous therapy
**Melanoma**								
MM1	53	M	Cutaneous	IIIc	LN	BRAF WT NRAS mutant	Nil	Adjuvant ipilimumab/nivolumab
MM2	52	M	Cutaneous	IV – M1c	Small bowel	BRAF mutant	Pembrolizumab	Ipilimumab
MM3	76	F	Cutaneous	IV – M1c	Small bowel	BRAF WT NRAS mutant	Nil	Nil
MM4	76	M	Cutaneous	IIIc	LN	BRAF mutant	Nil	Nil
MM5	74	F	Cutaneous	IV – M1a	LN	BRAF WT	Nil	Nil
MM6	61	M	Cutaneous	IV – M1a	LN	BRAF WT	Nil	Ipilimumab
MM7	42	M	Cutaneous	IIIc	LN	BRAF mutant	Nil	Nil
MM8	49	M	Cutaneous	IV – M1c	LN	BRAF WT NRAS mutant	Nil	Paclitaxel + Trametinib Ipilimumab Pembrolizumab
**NSCLC**								
NSCLC1 (CRUK0394)	74	M	Adenocarcinoma	IIa	Right lower lobe	-	Nil	Nil
NSCLC2 (CRUK0400)	76	M	Squamous	IIIa	Right upper lobe	-	Nil	Nil
NSCLC3 (CRUK0381)	53	F	Squamous	IIa	Left upper lobe	-	Nil	Nil
NSCLC4 (CRUK0403)	90	M	Squamous	IIa	Left lower lobe	-	Nil	Nil
NSCLC5 (CRUK0178)	60	M	Adenocarcinoma	IIa	Left upper lobe	-	Nil	Nil
NSCLC6 (CRUK0009)	57	M	Adenocarcinoma	IIb	Left lower Lobe	-	Nil	Nil
NSCLC7 (CRUK0230)	54	F	Adenocarcinoma	IIa	Right upper lobe	-	Nil	Nil
NSCLC8	69	M	Adenocarcinoma	Ia	Left lower lobe	-	Nil	Nil

Identifier	Age	Sex	Subtype	Stage	Biopsy/ resection site	Mutational status	Current therapy	Previous therapy
**RCC**								
RCC1	44	M	Clear cell	III	-	-	Nil	Nil
RCC2	69	M	Clear cell	IIa	-	-	Nil	Nil
RCC3	78	F	Mixed - clear cell/ tubulo-papillary	Ia	-	-	Nil	Nil
RCC4	65	F	Clear cell	IV	-	-	Nil	Nil
RCC5	50	F	Clear cell	IV	-	-	Nil	Nil
RCC6	60	F	Clear cell	IV	-	-	Nil	Nil
RCC7	19	F	Clear cell	IV	-	-	Nil	Nil
RCC8	59	M	Clear cell	IV	-	-	Nil	Nil

M, male; F, female; LN, lymph node; WT, wild type.

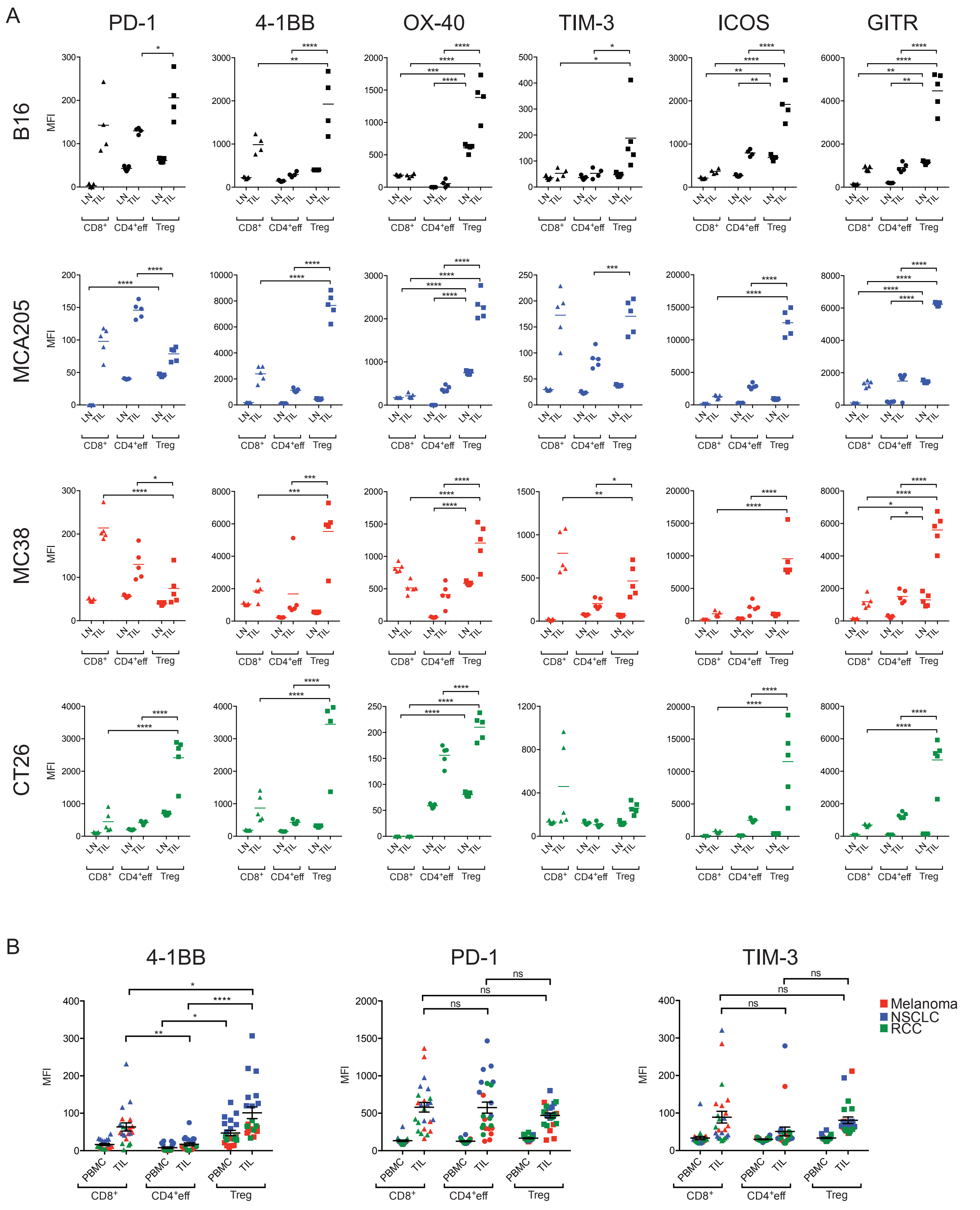

Figure S1, related to Figure 1. Expression profile of B7 and TNFR superfamily checkpoints on T cell subsets in murine and human tumors. (A) MFI of co-stimulatory and co-inhibitory molecules expressed on TIL subpopulations in murine tumors. (B) MFI of 4-1BB, PD-1 and TIM-3 expressed on TIL subpopulations in human tumors. Horizontal bars represent the mean and error bars show ± SEM; ns, no statistical significance.

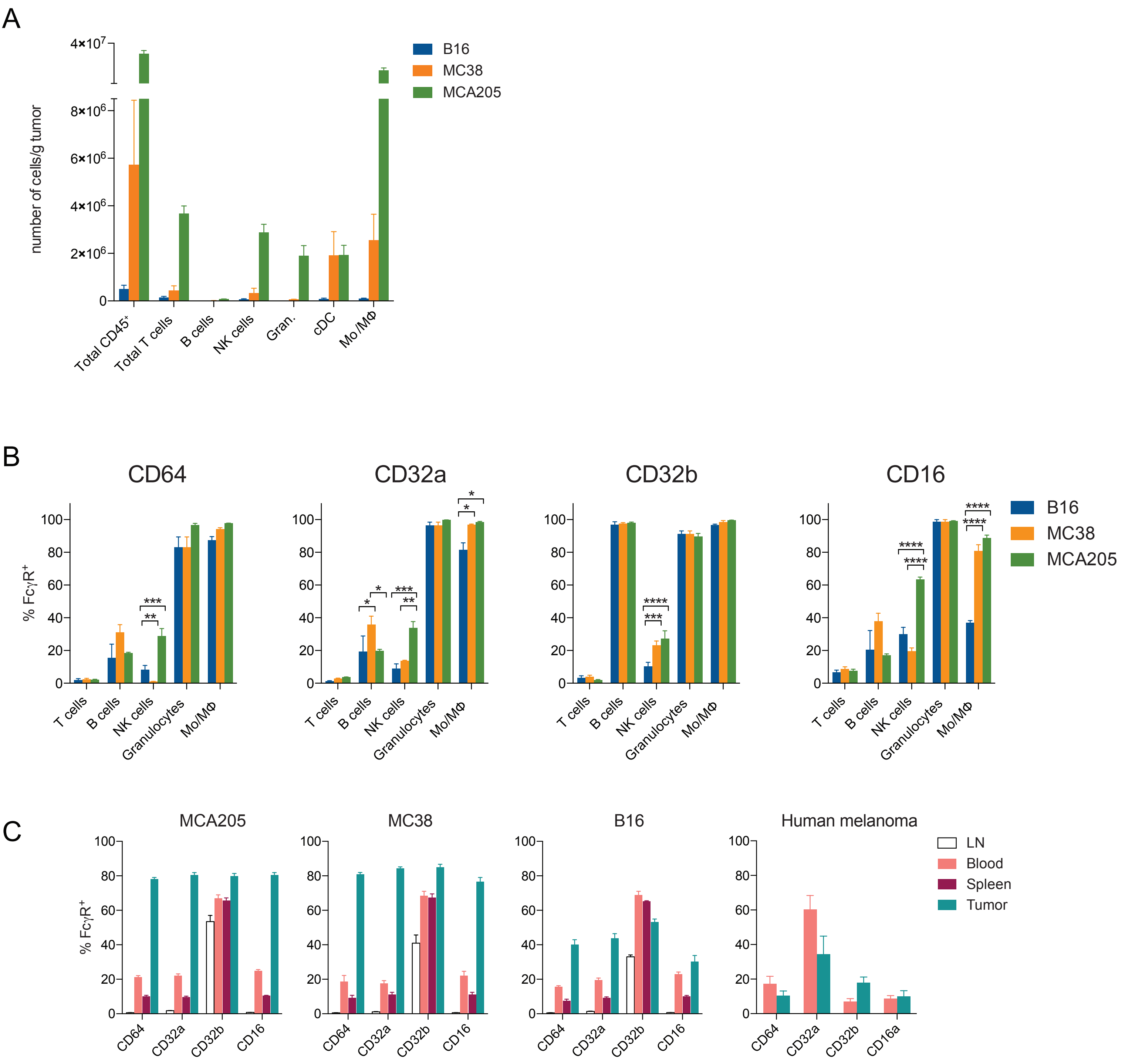

Figure S2, related to Figure 2. Expression profile of hFcγRs in hFcγR mice and human tumors. (A) Quantification of the absolute number of tumor-infiltrating leukocyte subpopulations in B16, MC38 and MCA205 tumors in hFcγR mice. (B) Percentage of expression of individual FcγRs in tumor-infiltrating leukocyte subpopulations in each tumor model. (C) Percentage of expression of individual FcγRs in total CD45+ cells from different organs and tissues in tumor-bearing mice or samples from patients with melanoma (see Table S2). Error bars show ± SEM.

Table S2, related to Figure 2. Demographics and clinical characteristics of patients with advanced malignant melanoma (MM) employed fresh for myeloid analyses.

Identifier	Age	Sex	Subtype	Stage	Biopsy site	Mutational status	Current therapy	Previous therapy
MM9	66	M	Cutaneous	IV M1c	LN	BRAF WT	Pembrolizumab	Ipilimumab
MM10	60	M	Ocular	IV M1c	Splenic metastasis	BRAF WT	Nil	Nil
MM11	76	M	Cutaneous	IV M1c	SC	BRAF mutant	Nil	Nil
MM12	86	F	Cutaneous	IV M1c	SC<br>LN	BRAF WT	Nil	Nil
MM13	46	F	Cutaneous	IV M1c	SC	BRAF mutant	Nil	Dabrafenib/Trametinib
MM14	50	M	Cutaneous	IV M1c	Small bowel	BRAF mutant	Pembrolizumab	Ipilimumab
MM15	69	M	Cutaneous	IV M1c	SC	BRAF WT	Pembrolizumab	Ipilimumab
MM16	76	F	Cutaneous	IV M1c	Small bowel	BRAF WT	Nil	Nil
MM17	53	M	Cutaneous	IIIc	LN	BRAF WT	Nil	Adjuvant ipilimumab/ nivolumab

M, male; F, female; LN, lymph node; SC, subcutaneous; WT, wild type.

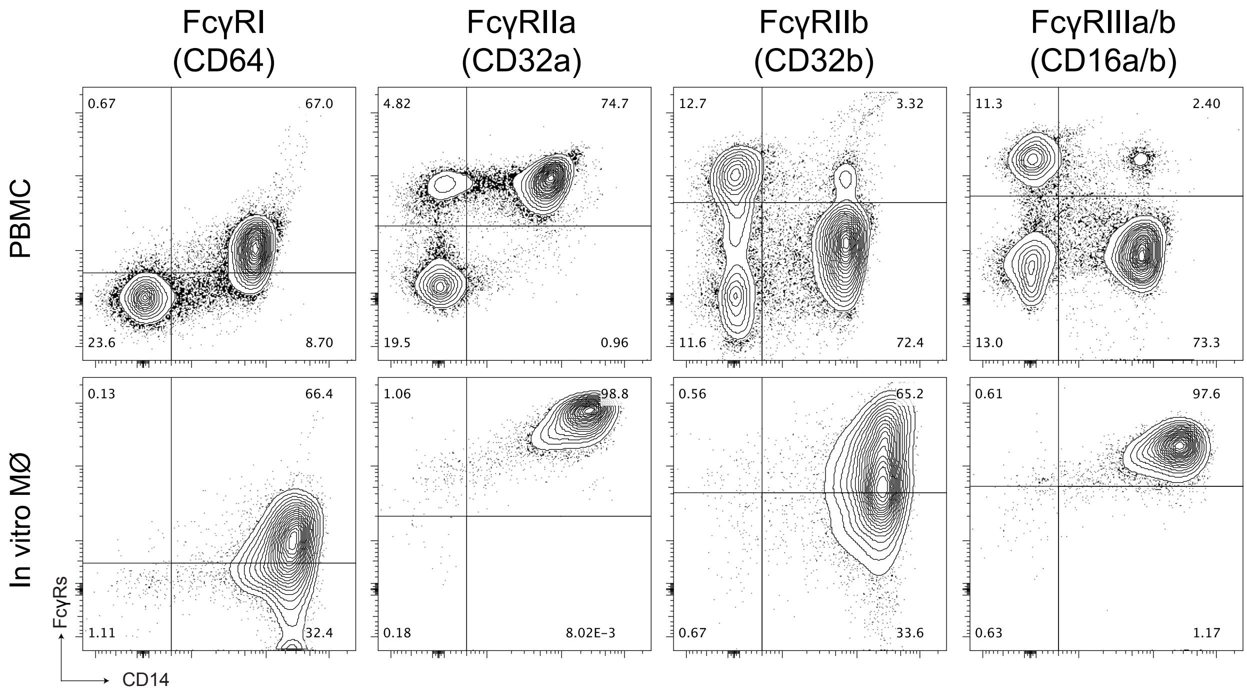

Figure S3, related to Figure 3. Expression pattern of FcγRs in monocyte-derived human macrophages. Expression of human FcγRs quantified by flow cytometry in $CD14^+$ bead-sorted monocytes from healthy donor PBMCs (upper panel) and matched macrophages (MΦ) seven days after in vitro differentiation with human recombinant M-CSF (lower panel).

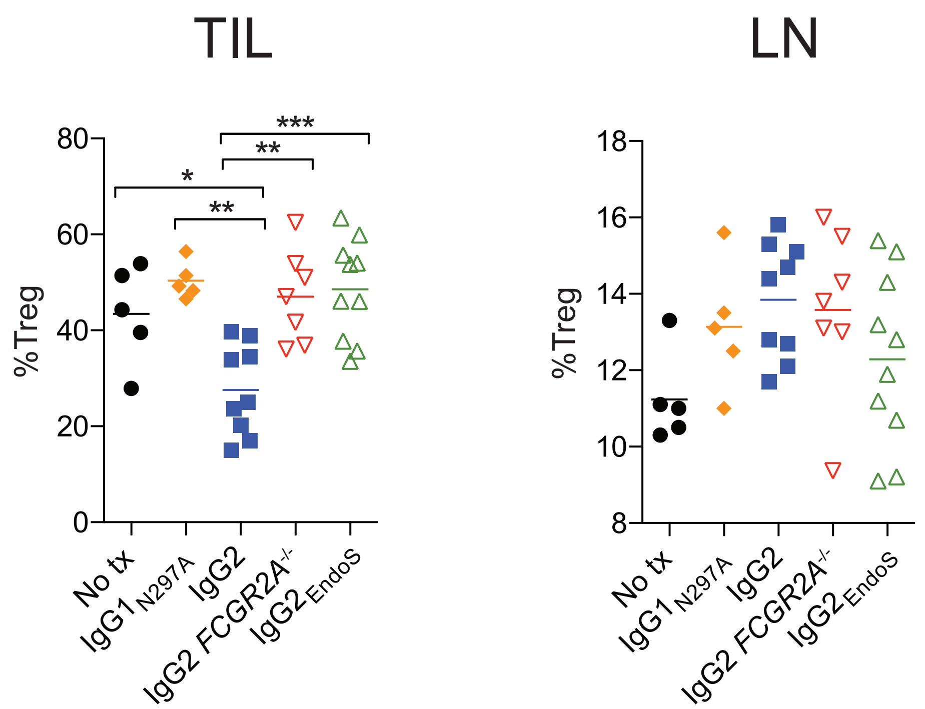

Figure S4, related to Figure 4. In vivo intra-tumoral Treg cell depletion by anti-CTLA-4-hIgG2 depends on CD32a. Percentage of $CD4^+FoxP3^+$ T cells of total $CD4^+$ T cells in TIL and LN in hFcγR mice bearing MCA205 tumors and treated with different IgG variants of anti-CTLA-4 mAbs as described in Figure 4. In the *FCGR2A*$^{-/-}$ group, mice expressed all human FcγRs except CD32a. $IgG2_{EndoS}$, endoglycosidase-treated IgG2 mAb. Horizontal bars show the mean.

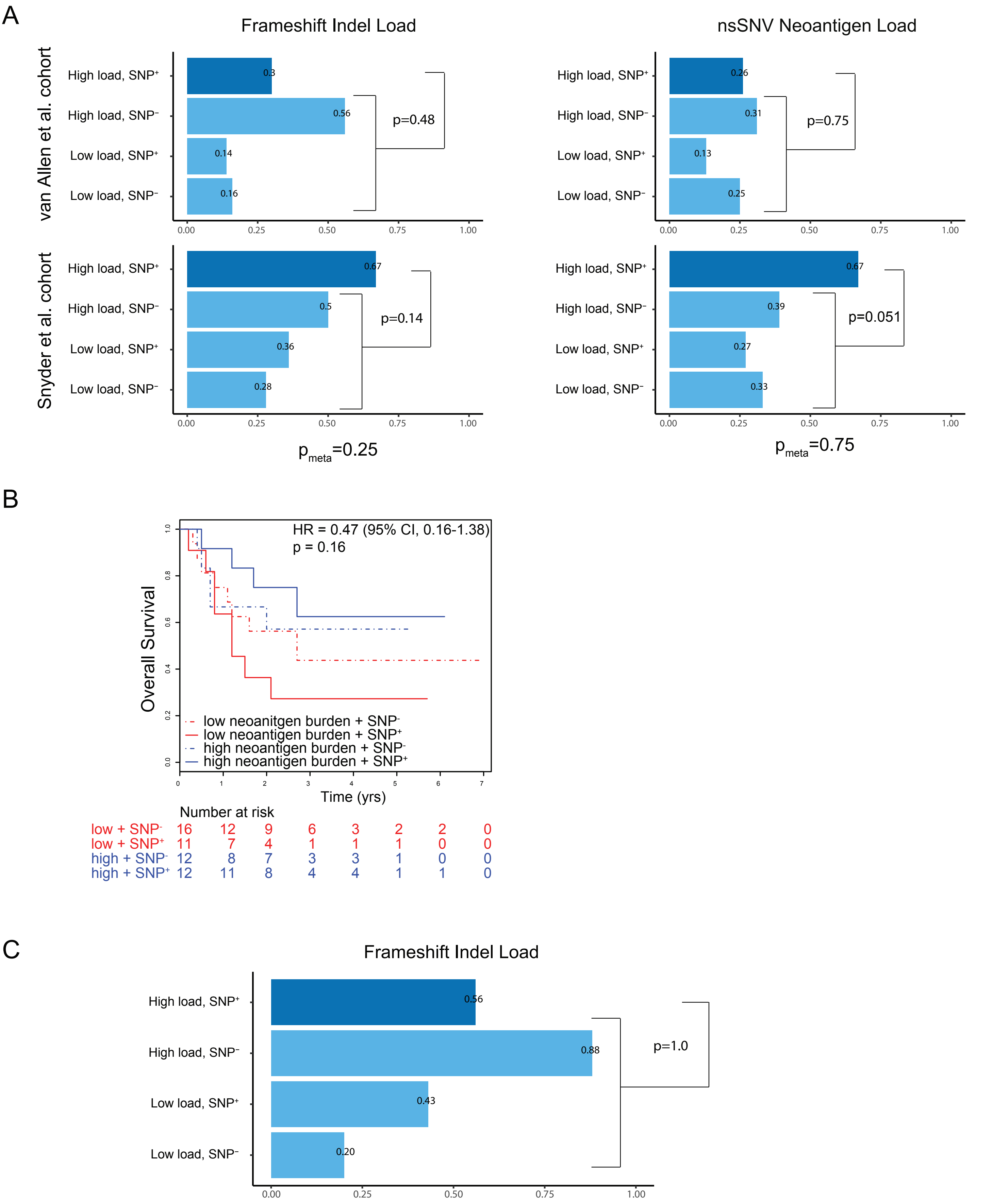

Figure S5, related to Figure 5. Human FcγR polymorphisms impact upon response to ipilimumab in patients with advanced melanoma. (A) Anti-CTLA-4 response rate in the van Allen et al. and Snyder et al. patient cohorts based on indel mutational load and nsSNV neoantigen load combined with the presence (SNP⁺) or absence (SNP⁻) of the germline CD32a-H131R high affinity SNP. (B) Survival analysis of the Snyder et al. patient cohort based on nsSNV neoantigen burden and germline CD32a-H131R SNP. (C) Response rate of patients treated with anti-PD-1 from the Hugo et al. dataset based on indel mutational load with (SNP⁺) or without (SNP⁻) the CD16-V158F high affinity SNP.
